# Vascular effects of serelaxin in patients with stable coronary artery disease: a randomized placebo-controlled trial

**DOI:** 10.1093/cvr/cvz345

**Published:** 2020-02-17

**Authors:** David Corcoran, Aleksandra Radjenovic, Ify R Mordi, Sheraz A Nazir, Simon J Wilson, Markus Hinder, Denise P Yates, Surendra Machineni, Jose Alcantara, Margaret F Prescott, Barbara Gugliotta, Yinuo Pang, Niko Tzemos, Scott I Semple, David E Newby, Gerry P McCann, Iain Squire, Colin Berry

**Affiliations:** 1 British Heart Foundation Glasgow Cardiovascular Research Centre, University of Glasgow, Glasgow, UK; 2 Golden Jubilee National Hospital, Glasgow, UK; 3 Department of Cardiovascular Sciences, University of Leicester and NIHR Leicester Biomedical Research Centre, Leicester, UK; 4 British Heart Foundation Centre for Cardiovascular Science, University of Edinburgh, Edinburgh, UK; 5 Novartis Institutes for Biomedical Research, Basel, Switzerland; 6 Novartis Institutes for BioMedical Research, Cambridge, MA, USA; 7 Novartis Healthcare Private Limited, Hyderabad, India; 8 Novartis Pharmaceuticals Corporation, East Hanover, NJ, USA; 9 London Health Science Centre, University of Western Ontario, London, Ontario, Canada

**Keywords:** Serelaxin, Coronary artery disease, Myocardial perfusion, Aortic stiffness

## Abstract

**Aims:**

The effects of serelaxin, a recombinant form of human relaxin-2 peptide, on vascular function in the coronary microvascular and systemic macrovascular circulation remain largely unknown. This mechanistic, clinical study assessed the effects of serelaxin on myocardial perfusion, aortic stiffness, and safety in patients with stable coronary artery disease (CAD).

**Methods and results:**

In this multicentre, double-blind, parallel-group, placebo-controlled study, 58 patients were randomized 1:1 to 48 h intravenous infusion of serelaxin (30 µg/kg/day) or matching placebo. The primary endpoints were change from baseline to 47 h post-initiation of the infusion in global myocardial perfusion reserve (MPR) assessed using adenosine stress perfusion cardiac magnetic resonance imaging, and applanation tonometry-derived augmentation index (AIx). Secondary endpoints were: change from baseline in AIx and pulse wave velocity, assessed at 47 h, Day 30, and Day 180; aortic distensibility at 47 h; pharmacokinetics and safety. Exploratory endpoints were the effect on cardiorenal biomarkers [N-terminal pro-brain natriuretic peptide (NT-proBNP), high-sensitivity troponin T (hsTnT), endothelin-1, and cystatin C]. Of 58 patients, 51 were included in the primary analysis (serelaxin, *n *=* *25; placebo, *n *=* *26). After 2 and 6 h of serelaxin infusion, mean placebo-corrected blood pressure reductions of −9.6 mmHg (*P *=* *0.01) and −13.5 mmHg (*P *=* *0.0003) for systolic blood pressure and −5.2 mmHg (*P *=* *0.02) and −8.4 mmHg (*P *=* *0.001) for diastolic blood pressure occurred. There were no between-group differences from baseline to 47 h in global MPR (−0.24 vs. −0.13, *P *=* *0.44) or AIx (3.49% vs. 0.04%, *P *=* *0.21) with serelaxin compared with placebo. Endothelin-1 and cystatin C levels decreased from baseline in the serelaxin group, and there were no clinically relevant changes observed with serelaxin for NT-proBNP or hsTnT. Similar numbers of serious adverse events were observed in both groups (serelaxin, *n *=* *5; placebo, *n *=* *7) to 180-day follow-up.

**Conclusion:**

In patients with stable CAD, 48 h intravenous serelaxin reduced blood pressure but did not alter myocardial perfusion.

## 1. Introduction

Serelaxin is a relaxin receptor agonist and recombinant form of the naturally occurring vasoactive human relaxin-2 peptide hormone.^1^ Relaxin plays a central role in haemodynamic and renal adaptations to pregnancy.^2^^,^^3^ Serelaxin mediates its effects through binding to its cognate receptor, relaxin/insulin-like family peptide receptor 1 (RXFP1). Serelaxin is pleiotropic with vasodilatory, anti-fibrotic, and end-organ protective effects.^4–6^ Pharmacological effects include increased nitric oxide and vascular endothelial growth factor production, inhibition of endothelin-1 and angiotensin II, and up-regulation of matrix metalloproteinases. These effects lead to endothelial-dependent arterial vasodilatation, increased arterial compliance, and other potentially favourable effects on cardiorenal haemodynamics.^5^^,^^6^

In the RELAX-AHF trial, intravenous (IV) serelaxin infusion was associated with an improvement in dyspnoea and a reduction in the risk of cardiovascular death at 6 months in patients hospitalized for acute heart failure (AHF).^1^ A subsequent larger trial, RELAX-AHF-2, reported neutral effects of serelaxin on in-hospital worsening of heart failure (HF) to Day 5 and on cardiovascular mortality at 180-day follow-up.^7^ Coronary artery disease (CAD) is the most common comorbidity in patients with AHF, and in the majority of patients CAD is the underlying aetiology of HF.^8^ The high prevalence of CAD in patients with AHF is evident in large clinical trial populations,^9–11^ including the RELAX-AHF and RELAX-AHF-2 cohorts (52% and 54%, respectively).^1^^,^^7^

Following RELAX-AHF, it was hypothesized that the beneficial effects on outcomes seen with serelaxin may be at least partly due to improvements in myocardial perfusion. Serelaxin may enhance coronary microvascular and endothelial function and lead to improved myocardial perfusion.^12^ Abnormal myocardial perfusion is a common finding in patients with both ischaemic and non-ischaemic cardiomyopathy and is prognostically relevant.^13–17^ Serelaxin may exert a cardioprotective effect in ischaemic–reperfusion injury, as may occur in patients with CAD and HF.^18^^,^^19^ Serelaxin may improve arterial compliance, and therefore myocardial perfusion, as coronary blood flow is dependent on aortic driving pressure and cardiac-coronary coupling.^20^^,^^21^ Increased aortic stiffness results in greater aortic reflected wave amplitude and aortic systolic blood pressure (SBP), thereby prolonging systole, reducing diastole and leading to reduced myocardial perfusion.^22^^,^^23^

Given its pleiotropic mechanisms, we hypothesized that serelaxin may enhance coronary microvascular and systemic macrovascular function. Conversely, coronary and systemic vasodilatation secondary to serelaxin may reduce coronary perfusion pressure, which may be clinically relevant and harmful in patients with obstructive CAD. This placebo-controlled mechanistic study investigated the acute effects of serelaxin on myocardial perfusion and aortic stiffness; circulating biomarkers of cardiorenal function in patients with established CAD; and overall safety to 180-day follow-up.

## 2. Methods

### 2.1 Study design

**Figure 1:**
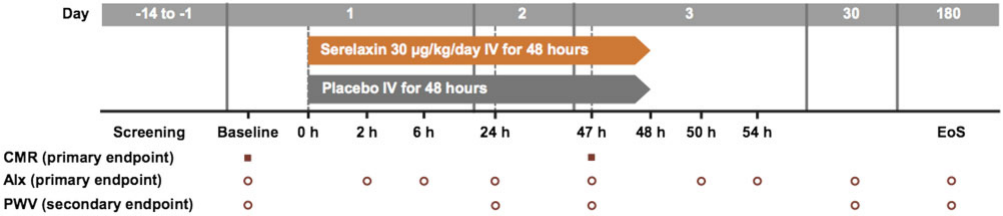
Study design and schedule for assessment of primary and secondary endpoints. AIx, augmentation index; CMR, cardiovascular magnetic resonance imaging; EoS, end of study; h, hours; IV, intravenous; PWV, pulse wave velocity.

This prospective, multicentre, double-blind, randomized, parallel-group, placebo-controlled, phase II study was conducted in three centres across the UK (ClinicalTrials.gov Identifier: NCT01979614) from February 2014 to February 2016. Patients were randomized 1:1 to a 48 h IV infusion of serelaxin

(30 μg/kg/day) or matching placebo, using a computerized randomization system involving concealed treatment arms. The co-primary endpoints were the change from baseline in global myocardial perfusion reserve (MPR) measured by quantitative perfusion cardiac magnetic resonance (CMR) imaging and the change from baseline in augmentation index (AIx) measured by applanation tonometry. The primary endpoints of MPR and AIx were assessed at baseline and 47 h post-commencement of the infusion (*Figure 1*).

Secondary endpoints included: the change from baseline in AIx and pulse wave velocity (PWV) assessed at the end of treatment, Day 30 and Day 180; aortic distensibility (assessed with CMR and PWV methods) at the end of treatment; pharmacokinetics and safety (adverse events, serious adverse events, and vital signs) of 48 h infusion of serelaxin 30 μg/kg/day. The changes in levels of circulating biomarkers of cardiorenal and vascular function during and after treatment with serelaxin were also assessed as part of the exploratory endpoints.

The study protocol and all amendments were reviewed by the Independent Ethics Committee. The study was conducted according to the ethical principles of the Declaration of Helsinki.

### 2.2 Participants

Male and female patients aged ≥18 years who had provided written informed consent were eligible for participation in this study. Inclusion criteria included proven obstructive CAD, either by functional testing (non-invasive ischaemia imaging) or anatomical imaging (invasive coronary angiography or computed tomography coronary angiography). Exclusion criteria included body weight ≥160 kg, acute coronary syndrome within 30 days prior to screening, known significant valvular heart disease, myocardial infarction <3 months, SBP <110 mmHg within 3 h prior to randomization, AHF at baseline, New York Heart Association (NYHA) Class III–IV HF, HF due to arrhythmia, women of child-bearing potential (unless using highly effective methods of contraception during dosing and for 3 months following study treatment), breast-feeding women, contraindications to CMR imaging, and severe renal impairment (estimated glomerular filtration rate < 30 mL/min/m^2^).

### 2.3 CMR acquisition and assessment

A standardized CMR protocol was acquired on 1.5 T (Siemens Avanto) or 3.0 T (Siemens Skyra and Verio) magnetic resonance imaging (MRI) scanners. The CMR protocol included standard localizers, long- and short-axis cine acquisitions, stress and rest first-pass perfusion, and late gadolinium enhancement (LGE) imaging as previously described.^24^ For perfusion imaging, a saturation recovery prepared fast gradient echo sequence was used to acquire dynamic contrast-enhanced CMR during adenosine stress (140–280 µg/kg/min for 3 min) and rest, during first-pass of the IV administered contrast agent (Gadovist, 0.05 mmol/kg, 5 mL/s), followed by 20 mL saline injection (5 mL/s). Three short-axis planes through the left ventricle (basal, mid-ventricular, and apical) were obtained.

Full left ventricular short-axis stack of cine images was acquired during the interval between stress and rest first-pass perfusion acquisitions. Following administration of the top-up contrast dose (Gadovist 0.05 mmol/kg), aortic cine and phase-velocity encoding sequences were acquired at the level of the pulmonary artery bifurcation. LGE imaging was performed with a segmented phase-sensitive inversion recovery sequence.

CMR image collection, blinding, and handling were managed by an independent imaging contract-research organization (CRO; VirtualScopics, Inc., Rochester, NY, USA). The CMR quantitative analyses were performed by blinded academic readers with >3 years of CMR experience (D.C., A.R., and C.B.). Cine images for ventricular volumes and mass and myocardial perfusion images were manually contoured (QMass, Medis, Leiden, Netherlands). Myocardial blood flow (MBF) was derived from stress and rest perfusion images using a Fermi deconvolution method,^25^ implemented in MATLAB.^26^ Global MPR was calculated from the ratio of global MBF evaluated at stress and at rest (MPR = stress MBF/rest MBF). Global MBF was calculated by averaging the MBF (mL/g/min) for basal, mid, and apical short-axis left ventricular slices. Ascending and descending aortic strain and distensibility were derived from manually contoured end-diastolic and end-systolic aortic cine images. Aortic strain (AS, unit free fraction) = [maximum aortic lumen area − minimum aortic lumen area]/minimum aortic lumen area. Aortic distensibility (AD, mmHg^−^^1^) = aortic strain/pulse pressure. Ascending aortic peak flow velocity (m/s) was derived from manually contoured phase-velocity encoding sequence images (QFlow, Medis, Leiden, Netherlands). The presence or absence of myocardial scar was qualitatively assessed on the LGE images.

### 2.4 Applanation tonometry assessments

The applanation tonometry assessments were performed by blinded operators. Augmentation index [AIx = (augmentation pressure/pulse pressure) × 100], and carotid-femoral PWV were measured by applanation tonometry (Sphygmocor^®^, AtCor Medical, Australia) at a proximal (carotid artery) and a distal (femoral artery) site of the body, utilizing R-wave gated determination of the time interval between the two measurement points. An averaged carotid pressure waveform (from a 10 s recording) was converted into a corresponding central waveform using a validated transfer function (SphygmoCor^®^, AtCor, Australia).^27^^,^^28^

### 2.5 Pharmacodynamics

Biomarkers reported include: N-terminal pro-brain natriuretic peptide (NT-proBNP), high-sensitivity troponin T (hsTnT) measured to assess myocardial safety; cystatin C measured to access renal function; the biologically active form of endothelin-1 (1–21), measured to assess vascular function. Venous blood samples were collected at 0 (pre-dose), 24, 48, and 54 h, Day 30, and Day 180. NT-proBNP, hsTnT, and cystatin C samples were collected in EDTA tubes while endothelin-1 samples were collected in EDTA tubes containing aprotinin. Plasma was immediately frozen at −70°C pending analysis in complete patient sets. Commercial biomarker kits were as follows: hsTnT from Roche Diagnostics GmbH, Germany; cystatin C from Gentian, Moss, Norway; and active endothelin-1 (1–22) from Biomedica Medizinprodukte, Vienna, Austria.

### 2.6 Pharmacokinetic profile and immunogenicity

Blood samples (approximately 2 mL) for pharmacokinetic (PK) evaluation were collected at 0 (pre-dose), 24, 48, 50, and 54 h, and Day 30. Serum serelaxin concentration was determined by an enzyme-linked immunosorbent assay, developed and validated based upon a commercially available kit (Quantikine ELISA kit, R&D systems, MN, USA).^29^ The PK parameters were calculated using non-compartmental methods that included steady-state concentration (Css) and systemic clearance (CL). Css was estimated using the serum concentration determined at 48 h, while CL was estimated using the rate of infusion and Css for each patient.

Blood was collected on Day 1 (baseline) and post-dose on Day 30 for assessment of immunogenicity. Serum anti-serelaxin antibody concentrations were evaluated using a validated four-tiered assay approach for antibody screening, confirmation, titration, and neutralizing antibody detection, when applicable.

### 2.7 Safety assessments

All adverse events (AEs) were recorded along with investigator-reported seriousness, intensity, and relationship to study drug. Clinical laboratory assessments (including haematology, biochemistry, and urinalysis), physical examination, electrocardiograms, and vital signs were assessed at Day 30 and Day 180.

### 2.8 Statistical analysis and sample size calculation

The change from baseline in mean global MBF (at rest and stress) and global MPR were analysed using analysis of covariance (ANCOVA) with treatment as the classification factor and baseline as a covariate, and by Bayesian approach assuming non-informative priors. The change from baseline in AIx and PWV were analysed using a repeated measures ANCOVA, including treatment, time, treatment by time, baseline by time interactions, and baseline as fixed effects. Time was repeated within each patient using an unstructured variance–covariance matrix. Adjusted mean difference between groups was provided and corresponding 95% confidence intervals (CIs) were calculated.

For the biomarker analyses, baseline was defined as the pre-dose assessment on Day 1. Raw values as well as changes from baseline were summarized by treatment and time. Descriptive statistics included geometric mean and 95% CI. For geometric means of change from baseline, a ratio to baseline was used to determine percentage change by first calculating in the log domain and then back transforming using exponentiation.

A priori power analysis indicated that data from 40 patients (20 per treatment group), would provide 83% power provided the true increase is at least 30%, or 64% power if the true increase is 25%. This estimate is based on an assumed mean MPR 2.48 [standard deviation (SD) 0.65], based on historical reference data. A sample size of 40 patients would have 80% power to detect a clinically significant difference of 10% (in AIx following 48 h serelaxin infusion) with a SD of 18 using a two-group *t*-test, with a one-sided test at a significance level of 5%.

## 3. Results

### 3.1 Study population

**Figure 2:**
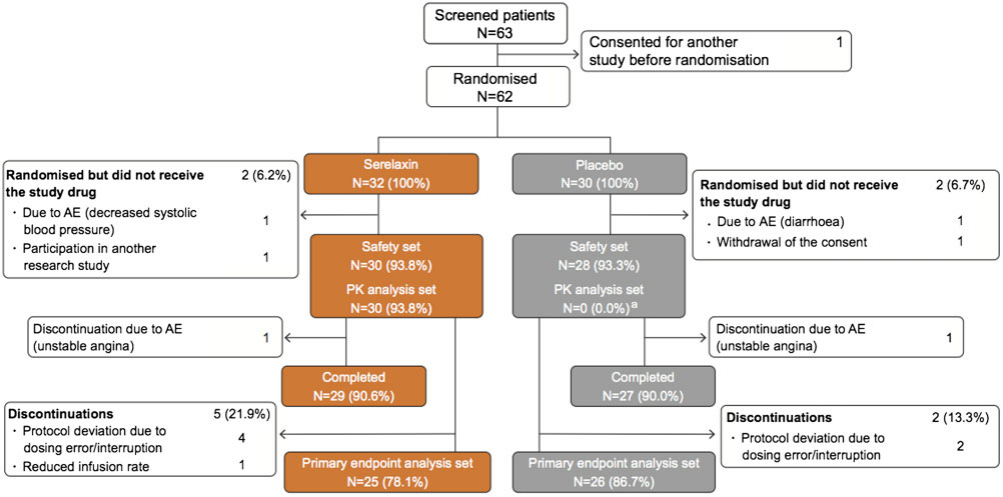
Study CONSORT diagram. AE, adverse event; PD, pharmacodynamic; PK, pharmacokinetic. ^a^Only the patients who received serelaxin were analysed and included in the PK analysis set.

A total of 62 patients were randomized to receive either serelaxin (*n* = 32) or placebo (*n* = 30), of which 58 received the study drug (serelaxin, *n* = 30; placebo, *n* = 28) and were included in the safety analysis set. Of 58 patients in the safety analysis set, 56 (97%) completed the study until Day 180 (serelaxin, *n* = 29; placebo, *n* = 27), and 51 (88%) were included in the primary outcome analysis (serelaxin, *n* = 25; placebo, *n* = 26). The baseline characteristics of patients by treatment group are provided in *Table 1* and the study CONSORT diagram is shown in *Figure 2*.

**Table 1:** Baseline characteristics

	Serelaxin	Placebo	All patients (dosed)
	(*N* = 30)	(*N* = 28)	(*N* = 58)
Demographics			
Age (years)	63 ± 6	60 ± 7	61 ± 7
Male (%)	27 (90)	26 (93)	53 (91)
Ethnicity (%)			
Caucasian	29 (97)	26 (93)	55 (95)
South Asian	1 (3)	1 (4)	2 (3)
Mixed ethnicity	0 (0)	1 (4)	1 (2)
Weight (kg)	90 ± 17	94 ± 15	92 ± 16
BMI (kg/m^2^)	30 ± 5	31 ± 4	30 ± 4
Medical history (%)			
Angina pectoris	18 (60)	18 (64)	36 (62)
Myocardial infarction	6 (20)	8 (29)	14 (24)
LV systolic dysfunction	2 (7)	0 (0)	2 (3)
Hypertension	17 (57)	19 (68)	36 (62)
Diabetes mellitus	6 (20)	4 (14)	10 (17)
Percutaneous coronary intervention	0 (0)	2 (7)	2 (3)
Cardiovascular medications (%)			
Anti-platelet drugs	30 (100)	28 (100)	58 (100)
Statin	29 (97)	28 (100)	57 (98)
Nitrate	26 (87)	25 (89)	51 (88)
Beta-blocker	25 (83)	24 (86)	49 (84)
ACEI	14 (47)	15 (54)	29 (50)
ARB	3 (10)	4 (14)	7 (12)
Diabetes medications (%)			
Metformin	5 (17)	7 (25)	12 (21)
DPP-4 inhibitors	0 (0)	4 (14)	4 (7)
Insulin and analogues	6 (20)	0 (0)	6 (10)
Baseline NT-proBNP^a^ (pg/mL)	87.0 (60.2–125.8)	81.5 (54.9–121.1)	84.4 (65.0–109.5)
Baseline CMR findings^b^			
LV EDV index (mL/m^2^)	82 ± 13	86 ± 16	84 ± 14
LV ESV index (mL/m^2^)	30 ± 8	32 ± 8	31 ± 8
LV mass index (g/m^2^)	39 ± 7	40 ± 9	39 ± 8
LV ejection fraction (%)	63 ± 5	64 ± 4	63 ± 4
RV EDV index (mL/m^2^)	71 ± 11	73 ± 12	72 ± 12
RV ESV index (mL/m^2^)	25 ± 4	26 ± 6	25 ± 5
RV ejection fraction (%)	65 ± 3	65 ± 4	65 ± 3
Patients with CMR LGE (%)	15 (30)	8 (29)	23 (40)

Data are presented as *n* (%) or mean ± SD where appropriate.

ACEI, angiotensin-converting enzyme inhibitors; ARB, angiotensin receptor blocker; BMI, body mass index; CAD, coronary artery disease; CMR, cardiac magnetic resonance; DPP-4, dipeptidyl peptidase 4; EDV, end-diastolic volume; ESV, end-systolic volume; LGE, late gadolinium enhancement; LV, left ventricular; NT-proBNP, N-terminal pro-brain natriuretic peptide; RV, right ventricular; SD, standard deviation.

a Data are presented as geometric mean (95% confidence intervals).

b These values correspond to the primary endpoint analysis set (serelaxin, *n *=* *25; placebo, *n *=* *26).

### 3.2 Primary endpoints

#### 3.2.1 Myocardial perfusion endpoints

**Figure 3:**
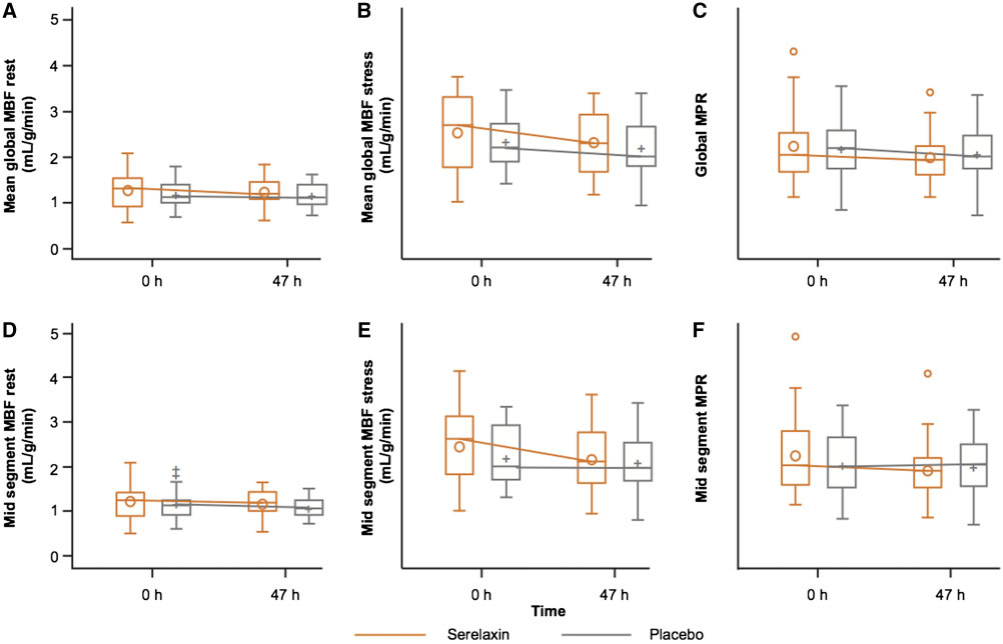
Absolute values of myocardial perfusion assessments at baseline and 47 h post-randomization in serelaxin vs. placebo groups. The box plots of the myocardial perfusion assessments were based on 51 patients (serelaxin, *n* = 25; placebo, *n* = 26) who were included in the pharmacodynamic analysis. (*A*) mean global rest MBF (*B*) mean global stress MBF (*C*) global MPR (*D*) mid segment rest MBF (*E*) mid segment stress MBF (*F*) mid segment MPR. h, hours; MBF, myocardial blood flow; MPR, myocardial perfusion reserve. Global MBF represents the mean MBF from all 16 American Heart Association (AHA) segments. Mid-MBF represents the mean MBF from the six mid-level left ventricular segments. The horizontal line in the box interior represents the median, while the symbol in the box interior represents the mean. Values outside the whiskers are identified with symbols and are extreme values. The upper (lower) edge of the box represents the 75th (25th) percentile. A whisker is drawn from the upper (lower) edge of the box to the largest (smallest) value within 1.5 interquartile range above (below) the edge of the box.

Compared with placebo, serelaxin infusion did not result in a significant change in the co-primary endpoint of change in MPR from baseline to 47 h [−0.24 vs. −0.13, adjusted mean difference −0.11; (95% CI −0.4 to 0.18); *P *=* *0.44]. No change was observed following serelaxin vs. placebo in mean MBF during stress [−0.11 (95% CI −0.45 to 0.22); *P *=* *0.76] or at rest [0.06 (95% CI −0.06 to 0.18); *P *=* *0.40], either globally or on an individual left ventricular slice level (*Figure 3* and Supplementary material online, *Tables S1*–*S3*).

#### 3.2.2 Augmentation index endpoints

**Figure 4:**
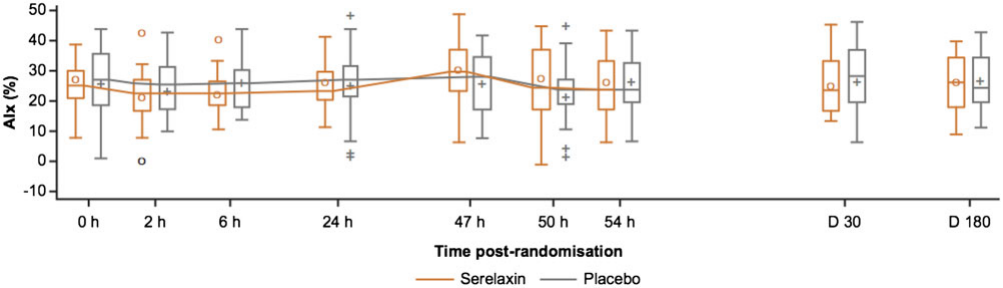
Absolute values of augmentation index in the serelaxin vs. placebo groups at study time points. The box plots of the augmentation index assessments were based on 51 patients (serelaxin, *n* = 25; placebo, *n* = 26) who were included in the final analysis. AIx, augmentation index; D, days; h, hours. The horizontal line in the box interior represents the median, while the symbol in the box interior represents the mean. Values outside the whiskers are identified with symbols and are extreme values. The upper (lower) edge of the box represents the 75th (25th) percentile. A whisker is drawn from the upper (lower) edge of the box to the largest (smallest) value within 1.5× interquartile range above (below) the edge of the box.

Compared with the placebo group, no significant differences in AIx were observed following serelaxin treatment at 47 h [3.49% vs. 0.04%, adjusted mean difference 3.45 (95% CI −2.04 to 8.95), *P *=* *0.21]. No change was observed following serelaxin vs. placebo at 2 h [serelaxin vs. placebo adjusted mean difference −2.12 (95% CI −7.49 to 3.25); *P *=* *0.43], 6 h [−2.88 (95% CI −6.84 to 1.08); *P *=* *0.15], 24 h [–0.84 (95% CI −5.38 to 3.7); *P *=* *0.71], 50 h [3.25 (95% CI −2.90 to 9.41); *P *=* *0.29], 54 h [−0.98 (95% CI −6.72 to 4.75); *P *=* *0.73], Day 30 [−1.75 (95% CI −7.26 to 3.75); *P *=* *0.52], or Day 180 [−0.05 (95% CI −4.67 to 4.57); *P *=* *0.98] (*Figure 4* and Supplementary material online, *Tables S4* and *S5*).

### 3.3 Secondary endpoints

#### 3.3.1 Aortic stiffness

Compared with the placebo group, treatment with serelaxin resulted in no difference in PWV at 24 h [serelaxin vs. placebo adjusted mean difference −0.16 (95% CI −0.86 to 0.54); *P *=* *0.65], 47 h [−0.34 (95% CI −1.24 to 0.56); *P *=* *0.45], Day 30 [−0.06 (95% CI −0.97 to 0.86); *P *=* *0.9], or Day 180 [0.12 (95% CI −0.83 to 1.06); *P *=* *0.80] (Supplementary material online, *Figure S1* and *Tables S6* and *S7*).

There was no significant change (*P *>* *0.05 for all) from baseline to 47 h in the serelaxin group compared with placebo in the CMR-derived parameters of aortic stiffness (ascending aorta strain, ascending aorta distensibility, descending aorta strain, descending aorta distensibility, and ascending aortic peak flow velocity) (Supplementary material online, *Figure S2* and *Tables S8A* and *B*).

#### 3.3.2 Left and right ventricular volumes and function

There were no statistically significant changes from baseline in the serelaxin group compared with placebo in the left and right ventricular volumes and ejection fractions. The left ventricular ejection fraction remained in the normal range (mean value 63%) with no significant changes observed with serelaxin treatment (Supplementary material online, *Figures S3* and *S4*).

#### 3.3.3 Pharmacodynamics

A summary of the biomarker analysis is shown in Supplementary material online*Table S9*.

The geometric means of hsTnT at baseline were 7.6 ng/L in the serelaxin group and 7.1 ng/L in the placebo group. A statistically significant decrease in hsTnT from baseline was observed following treatment with serelaxin at 48 h (geometric mean ratio 0.87; 95% CI 0.79–0.95). No statistically significant change from baseline was observed with serelaxin at 24 h and 54 h or in the placebo group (*P *>* *0.05).

The geometric means of cystatin C at baseline were 0.93 mg/L in the serelaxin group and 0.87 mg/L in the placebo group. A statistically significant decrease in cystatin C from baseline was observed following treatment with serelaxin at 24 h (cystatin C geometric mean ratio 0.92; 95% CI 0.89–0.94, 48 h (geometric mean ratio 0.93; 95% CI 0. 91–0.96), and 54 h (geometric mean ratio 0.87; 95% CI 0.80–0.94). The change in cystatin C levels from baseline in the placebo group were not statistically significant at 24 and 48 h, but a statistically significant change was observed at 54 h (geometric mean ratio 0.95; 95% CI 0.91–0.99).

The geometric means of endothelin-1 at baseline were 0.874 pmol/L in the serelaxin group and 0.771 pmol/L in the placebo group. Endothelin-1 levels were significantly decreased from baseline in the serelaxin group at 24 h (geometric mean ratio 0.82; 95% CI 0.68–0.99), 48 h (geometric mean ratio 0.82; 95% CI 0.70–0.96), and 54 h (geometric mean ratio 0.84; 95% CI 0.73–0.96). No statistically significant endothelin-1 change from baseline was observed in the placebo group.

The geometric means of NT-proBNP at baseline were 87.0 pg/mL in the serelaxin group and 81.5 pg/mL in the placebo group. NT-proBNP levels were statistically significantly increased from baseline in the serelaxin group at 54 h (geometric mean ratio 1.36; 95% CI 1.06–1.75) to a level of 117.1 pg/mL but were not statistically significantly changed at 24 h or 48 h. In the placebo group, NT-proBNP levels statistically significantly decreased compared with baseline at 24 h (geometric mean ratio 0.78; 95% CI 0.69–0.88) and 48 h (geometric mean ratio 0.76; 95% CI 0.60–0.95) with no statistically significant change at 54 h.

#### 3.3.4 Pharmacokinetics and immunogenicity

Following a 48 h IV infusion of serelaxin, geometric mean serum concentration of serelaxin increased gradually from 24 to 48 h during the IV infusion, followed by a rapid decline after the infusion stopped at 48 h (Supplementary material online, *Figure S5*). Serelaxin serum concentrations at 24 h were similar to those at 48 h (prior to end of infusion), indicating that steady state was achieved. The geometric means for the estimated Css and CL were 15.1 ng/mL and 83.0 mL/h/kg, respectively.

Immunogenicity analysis demonstrated that all patients were anti-serelaxin antibody negative prior to dosing on Day 1 (baseline) and at follow-up on Day 30.

#### 3.3.5 Safety endpoints

After 2 and 6 h of serelaxin infusion, mean placebo-corrected blood pressure reductions of approximately −9.6 mmHg (*P *=* *0.01) and −13.5 mmHg (*P *=* *0.0003) for SBP and −5.2 mmHg (*P *=* *0.02) and −8.4 mmHg (*P *=* *0.001) for diastolic blood pressure (DBP) were observed (Supplementary material online, *Figure S6* and *Table S10*). No patient discontinued the study due to hypotensive events.

None of the AEs reported during this study (serelaxin, *n* = 17; placebo, *n* = 19) were considered to be causally related to serelaxin (Supplementary material online, *Table S11*). The incidence of serious AEs (SAEs) was similar between groups (serelaxin, *n* = 5; placebo, *n* = 7). Three patients (serelaxin, *n* = 1; placebo, *n* = 2) experienced an SAE during study drug infusion (all unstable angina). Two patients (serelaxin, *n* = 1; placebo, *n* = 1) discontinued from the study drug infusion due to an SAE (unstable angina and non-ST elevation myocardial infarction). A relationship between the event and the study medication was not suspected. One placebo-treated patient had an SAE of moderate intensity (type IV hypersensitivity reaction, allergic dermatitis), which was suspected to be treatment related. The SAEs (angina pectoris, unstable angina, acute myocardial infarction, angio-oedema, gastro-oesophageal reflux, pleural effusion, vascular procedure-related complication, and cardiac procedure-related complication) reported for the remaining nine patients (serelaxin, *n* = 4; placebo, *n* = 5) were severe in intensity, but not suspected to be study drug related. No deaths were reported during this study.

## 4. Discussion

This study investigated the effects of serelaxin on coronary microvascular and systemic macrovascular function in patients with stable CAD, the single most common comorbidity in patients with AHF.^9–11^ In this study, 48 h of intravenous serelaxin reduced blood pressure but did not alter myocardial perfusion or aortic stiffness. Myocardial perfusion was maintained despite a reduction in arterial blood pressure, and serelaxin was well-tolerated.

In contrast to RELAX-AHF and RELAX-AHF-2, we enrolled patients with stable CAD and excluded patients with AHF at baseline or NYHA Class III–IV HF at baseline. The presence of obstructive CAD leading to ischaemia is an adverse prognostic factor in patients with AHF, as reflected by elevated serum concentrations of troponin and worse clinical outcomes.^30^ The benefits of IV serelaxin infusion that were observed in the RELAX-AHF trial^1^ were considered to be potentially mediated through improvements in myocardial perfusion leading to a reduction in myocardial ischaemia in patients with AHF. Conversely, should an IV infusion of serelaxin reduce arterial blood pressure in patients with obstructive CAD, and if coronary autoregulation is insufficient, myocardial perfusion may be reduced, potentially provoking harmful ischaemia.

No serelaxin-induced changes in coronary microvascular function were observed. The observed stress-induced mean increase in global MBF was ∼1 mL/g/min. This is lower in comparison to healthy control cohorts and reflects the underlying CAD in the study population.^31–33^ The lack of effect of serelaxin on mean global MBF and MPR is considered to be scientifically interesting, particularly in light of previous observations with nitroprusside and dipyridamole,^34^^,^^35^ that have been associated with reduced local myocardial perfusion, particularly in redistribution of blood away from ischaemic regions of the heart.^34^^,^^35^

Aortic stiffness is associated with an increased risk of developing HF^36^ and is abnormal in patients with HF.^37^ In our study, parameters of macrovascular function measured with applanation tonometry (AIx and PWV) and CMR (aortic distensibility, strain, and peak flow velocity) methods revealed no change following treatment with serelaxin. However, following 2 and 6 h of serelaxin infusion, mean placebo-corrected blood pressure reductions of approximately −9.6 and −13.5 mmHg for SBP, and −5.2 and −8.4 mmHg for DBP were observed. No difference in SBP or DBP was observed between 24 and 48 h post-infusion. These blood pressure changes did not translate into detectable changes in MBF as assessed by perfusion CMR.

We did not detect an improvement in MPR or AIx with the serelaxin dose and duration 48 h administered in the RELAX-AHF and RELAX-AHF-2 trials. The pathophysiological and haemodynamic mechanisms of AHF may occur over many weeks. It is plausible that a 48 h serelaxin infusion may be too short to recruit an effect on coronary microvascular and systemic macrovascular function. Prior pre-clinical studies demonstrating improved vascular function and anti-fibrotic benefits have used prolonged dosing protocols.^38–41^ In our study, the mean left ventricular ejection fraction was 63%, whereas it was 39% in the RELAX-AHF cohort, reflecting the different populations enrolled, and it is plausible that this may have influenced the results. Finally, the neutral result may be attributed in part to the effects of baseline vasodilating medications in this specific patient population. The PK analysis provides supporting evidence that the lack of an effect of serelaxin on MPR and AIx is not due to a lack of PK exposure. The RXFP1 has been demonstrated in cardiac tissue and cardiomyocytes.^19^^,^^42^^,^^43^ Consistent with previous observations, typical serum PK serelaxin concentrations (an additional quality control measure) were observed over the 48 h infusion period, with steady-state achieved between 24 and 48 h, with a rapid decline post-infusion thereafter.^44^^,^^45^

No clinically relevant change from baseline in hsTnT was observed in either treatment group, supporting myocardial safety in both treatment groups. A significant reduction in hsTnT was observed following 48 h serelaxin infusion vs. placebo, although levels were still considered to be in the normal range and this difference did not persist at 54 h. The cystatin C reductions observed with serelaxin at 24, 48, and 54 h are consistent with the renal benefit previously observed in patients with AHF.^46^ The reduction in endothelin-1 with serelaxin at 24, 48, and 54 h suggests beneficial effects on vascular function, consistent with improvement in biomarkers related to cardiac, renal, and hepatic damage previously observed in patients with AHF.^46^ These observations reflect the mechanism of action of serelaxin, which has been shown to increase expression of the endothelin B receptor, thereby acting as a sink to endothelin-1.^47^ There was a small but statistically significant increase in NT-proBNP from baseline to 54 h in the serelaxin group. No significant changes were observed at any other time points and the clinical relevance of this finding is uncertain.

The safety observation period in this study following the 48 h infusion extended until Day 180. No deaths occurred during the study period, and the numbers of SAEs reported in each treatment group were similar. The frequency and intensity of AEs were comparable between groups, with no new safety findings identified for serelaxin.

This study is limited in that myocardial relaxin receptor (RXFP1) expression remains unknown in patients with cardiovascular disease, leaving the potential to improve myocardial perfusion uncertain. CMR imaging was performed on different types of MRI scanners and field strengths across participating centres, implying some differences in measurement sensitivity in dynamic imaging of myocardial perfusion. However, this CMR measurement limitation is not supported by the observation of stable resting blood flow measurements in both placebo- and serelaxin-treated groups. The left ventricular haemodynamic data were consistent across sites and field strengths and any potential variations in analyses were controlled by central blinded reading. The methods of image analysis were the same, independent of MRI scanner type. We assessed the effects of serelaxin on myocardial perfusion during systemic hyperaemia induced by IV infusion of adenosine. Serelaxin acts via an endothelial-dependent mechanism that is distinct from adenosine, which acts via an endothelial-independent mechanism.^48^ We cannot discount the possibility that the effect of serelaxin on myocardial perfusion with adenosine pharmacological stress may have been blunted, and therefore, we may have had limited sensitivity to detect any treatment effect. We assessed global MBF by CMR imaging and did not align the myocardial perfusion data with the perfusion territory of the major epicardial coronary arteries. Investigating the effect of serelaxin on myocardial perfusion in myocardial segments subtended by a coronary artery with a flow-limiting stenosis compared to segments with normal epicardial blood flow may have been informative.

In conclusion, intravenous administration of serelaxin (30 µg/kg/day) for 48 h in patients with stable CAD did not affect myocardial perfusion or aortic stiffness, despite a mild lowering of SBP. No clinically relevant changes were observed with serelaxin for NT-proBNP or hsTnT.

## Supplementary material

Supplementary material is available at *Cardiovascular Research* online.

## Authors’ contributions

D.C.: coordinated the study at the Glasgow site, including participating in patient recruitment, obtaining informed consent in the participants, administering the study protocol, and collecting the clinical data. He led the CMR analysis, and interpreted the results. He jointly wrote the first draft of the manuscript. A.R.: led the CMR perfusion analysis, interpreted the data and contributed to the manuscript. I.M., S.A.N., and S.J.W.: coordinated the study at their study sites (Glasgow, Leicester, and Edinburgh, respectively) including participating in patient recruitment, obtaining informed consent, administering the study protocol, and collecting the clinical data. They interpreted the data and contributed to the manuscript. I.M. also contributed to the research ethics approvals. D.P.Y.: (Director of Clinical and Translational Imaging, Novartis) coordinated the CMR imaging analysis, interpreted the data and contributed to the manuscript. M.H.: (Executive Director Translational Medicine, Novartis) conceived the idea for the study, coordinated the study sites, interpreted the data and contributed to the manuscript. S.M.: (Principal Biostatistician, Novartis) led the statistical analyses, interpreted the data and contributed to the manuscript. M.F.P.: (Principal Medical Scientific Expert, Novartis) led the biomarker analysis, interpreted the data and contributed to the manuscript. B.G. and J.A.: (Clinical Trial Leads, Novartis) coordinated the study protocol at the three study sites, interpreted the data and contributed to the manuscript. Y.P.: (PK/PD Expert, Novartis) led the pharmacokinetics and pharmacodynamics analyses, interpreted the data and contributed to the manuscript. N.T., S.I.S., D.E.N., G.P.M., and I.S.: principal investigators at the study sites. They co-ordinated the delivery of the trial at the study sites, including participating in patient recruitment. They interpreted the data and contributed to the manuscript. G.P.M. and I.S. also developed the study protocol. N.T. and G.P.M. wrote the CMR standard operating procedure/Imaging protocol. C.B.: chief investigator for the study. C.B. contributed to the idea for the study, helped develop the protocol and managed the research ethics. He participated in patient recruitment, collected clinical data, interpreted the results, and jointly wrote the first draft of the manuscript.

## Supplementary Material

cvz345_Supplementary_Data

## Notes

**Time for primary review: 28 days**

## Notes

Translational perspective This multicentre, double-blind, parallel-group, placebo-controlled study showed that in patients with stable CAD, serelaxin did not affect myocardial perfusion or aortic stiffness. This was despite a mild lowering of systolic blood pressure with serelaxin therapy. The lack of a detectable effect of serelaxin on myocardial perfusion is clinically relevant, as no reduction in myocardial blood flow was found. Serelaxin, in addition to concomitant standard of care medication for stable CAD, was overall well-tolerated in the study participants.
