# Supplementary material for: Vascular effects of serelaxin in patients with stable coronary artery disease: a randomized placebo-controlled trial
Source: Cardiovasc Res. 2020 Feb 17;117(1):320–9. doi: 10.1093/cvr/cvz345 (PMC7797213; doi:10.1093/cvr/cvz345)
Supplement: cvz345_Supplementary_Data [file cvz345_supplementary_data.zip › cvz345-suppl_data/A2203_supplemental material_ESC CV Research_2019-11-20.docx]

# Supplementary Material

**Vascular effects of serelaxin in patients with stable coronary artery disease: a randomised placebo-controlled trial**

David Corcoran^1^, Aleksandra Radjenovic^1^, Ify Mordi^1^, Sheraz A. Nazir^2^, Simon J. Wilson^3^, Markus Hinder^4^, Denise P. Yates^7^, Surendra Machineni^6^, Jose Alcantara^4^, Margaret F. Prescott^5^, Barbara Gugliotta^4^, Yinuo Pang^7^, Niko Tzemos^8^, Scott I. Semple^3^, David E. Newby^3^, Gerry P. McCann^2^, Iain Squire^2^, and Colin Berry^1*^

**Affiliations:**

^1^British Heart Foundation Glasgow Cardiovascular Research Centre, University of Glasgow, and Golden Jubilee National Hospital, Glasgow, UK; ^2^Department of Cardiovascular Sciences, University of Leicester and NIHR Leicester Biomedical Research Centre, Leicester, UK; ^3^British Heart Foundation Centre for Cardiovascular Science, University of Edinburgh, UK; ^4^Novartis Institutes for Biomedical Research, Basel, Switzerland; ^5^Novartis Pharmaceutical Corporation, East Hanover, NJ, USA; ^6^Novartis Healthcare Private Limited, Hyderabad, India; ^7^Novartis Institutes for Biomedical Research, Cambridge, MA, USA; ^8^London Health Science Centre, University of Western Ontario, London, Ontario

*Corresponding author. Telephone: +44 (0) 141 11330 1671 or +44 (0) 141 951 5180, Fax: +44 (0) 141 330 6794, Email: [colin.berry@glasgow.ac.uk](mailto:colin.berry@glasgow.ac.uk)

# Supplementary Figure Legends

**Supplementary Figure S1. Absolute values of PWV in the serelaxin versus placebo study timepoints.**

D, days; h, hours; PWV, pulse wave velocity. The horizontal line in the box interior represents the median, while the symbol in the box interior represents the mean. Values outside the whiskers are identified with symbols and are extreme values. The upper (lower) edge of the box represents the 75th (25th) percentile. A whisker is drawn from the upper (lower) edge of the box to the largest (smallest) value within 1.5x interquartile range above (below) the edge of the box.

**Supplementary Figure S2. Measurement of arterial stiffness parameters at baseline and
47 h using aortic MRI (serelaxin versus placebo).**

h, hours; MRI, magnetic resonance imaging. The horizontal line in the box interior represents the median, while the symbol in the box interior represents the mean. Values outside the whiskers are identified with symbols and are extreme values. The upper (lower) edge of the box represents the 75th (25th) percentile. A whisker is drawn from the upper (lower) edge of the box to the largest (smallest) value within 1.5x interquartile range above (below) the edge of the box.

**Supplementary Figure S3. Box plots of LV volumes and ejection fraction at baseline and
47 h post-randomisation in serelaxin versus placebo groups.**

h, hours; LV, left ventricle. The horizontal line in the box interior represents the median, while the symbol in the box interior represents the mean. Values outside the whiskers are identified with symbols and are extreme values. The upper (lower) edge of the box represents the 75th (25th) percentile. A whisker is drawn from the upper (lower) edge of the box to the largest (smallest) value within 1.5x interquartile range above (below) the edge of the box.

**Supplementary Figure S4. Box plots of RV volumes and ejection fraction at baseline and
47 h post-randomisation in serelaxin versus placebo groups.**

h, hours; RV, right ventricle. The horizontal line in the box interior represents the median, while the symbol in the box interior represents the mean. Values outside the whiskers are identified with symbols and are extreme values. The upper (lower) edge of the box represents the 75th (25th) percentile. A whisker is drawn from the upper (lower) edge of the box to the largest (smallest) value within 1.5x interquartile range above (below) the edge of the box.

**Supplementary Figure S5. Geometric mean serelaxin serum PK concentration-time profiles.**

PK, pharmacokinetics

**Supplementary Figure S6. Measurement of vital signs (blood pressure and pulse rate) over time in both serelaxin and placebo groups.**

BP, blood pressure; D, day(s). The horizontal line in the box interior represents the median, while the symbol in the box interior represents the mean. Values outside the whiskers are identified with symbols. The upper (lower) edge of the box represents the 75th (25th) percentile. A whisker is drawn from the upper (lower) edge of the box to the largest (smallest) value with 1.5x interquartile range above (below) the edge of the box.

# Figures

**Figure S1**

**
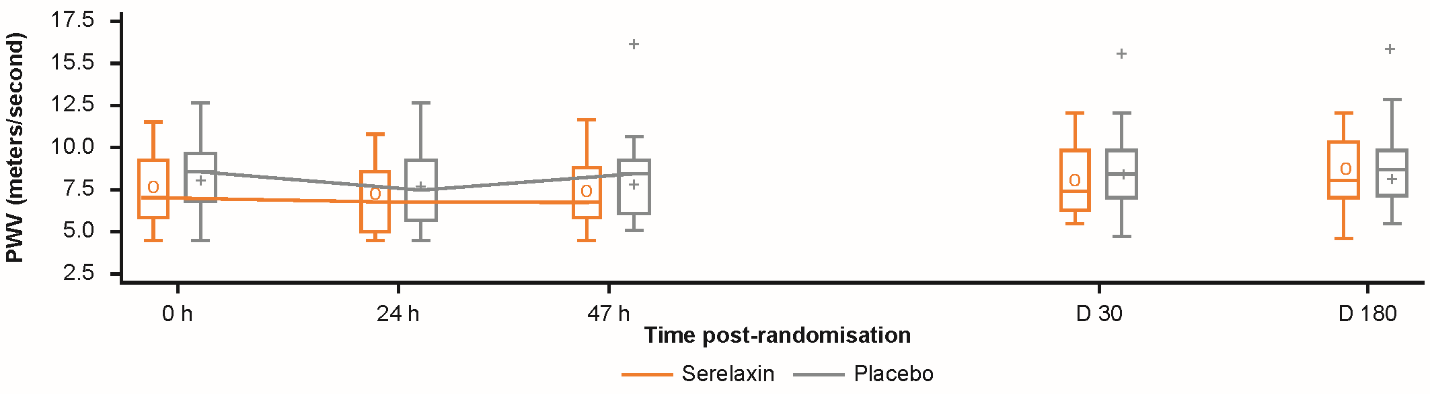
**

**Figure S2**

**
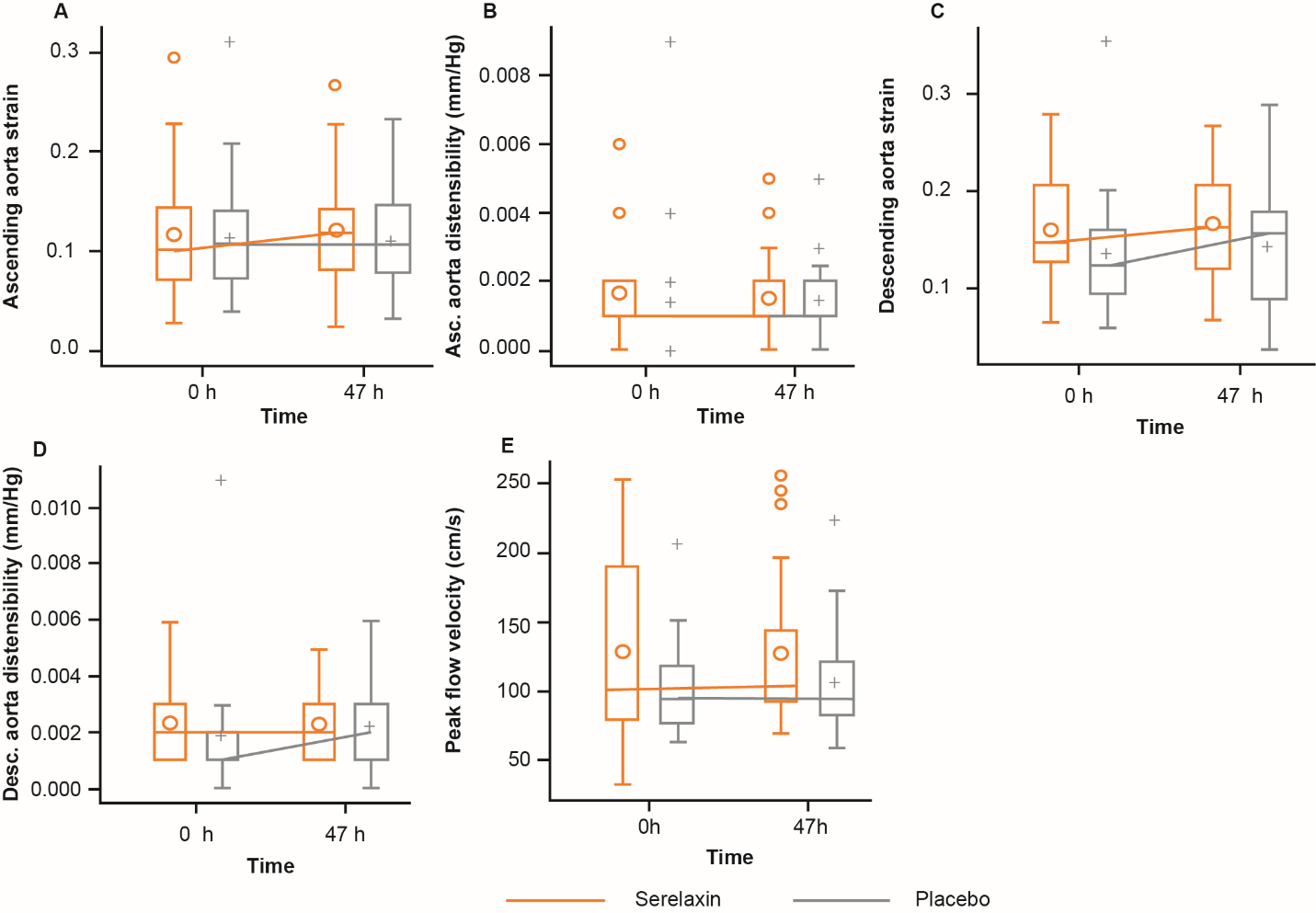
**

**Figure S3**

**
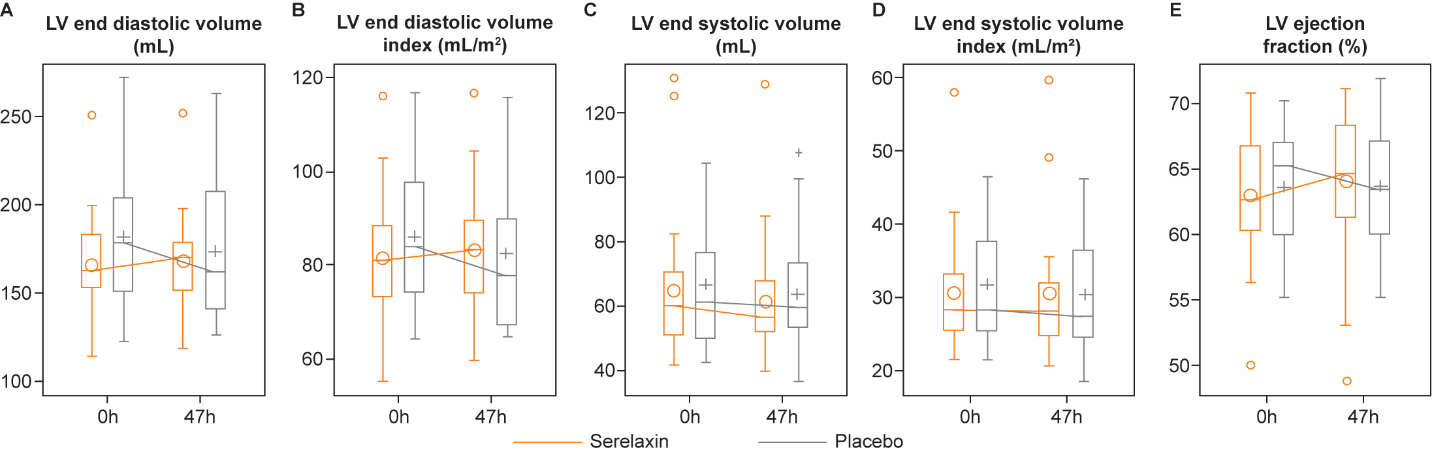
**

**Figure S4**

**
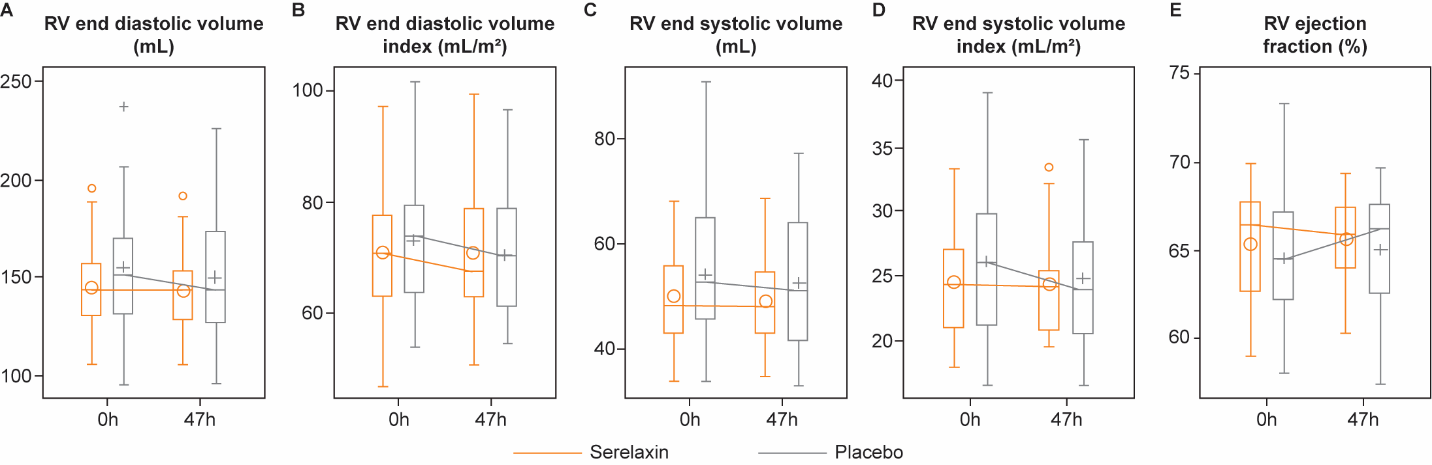
**

**Figure S5**

**
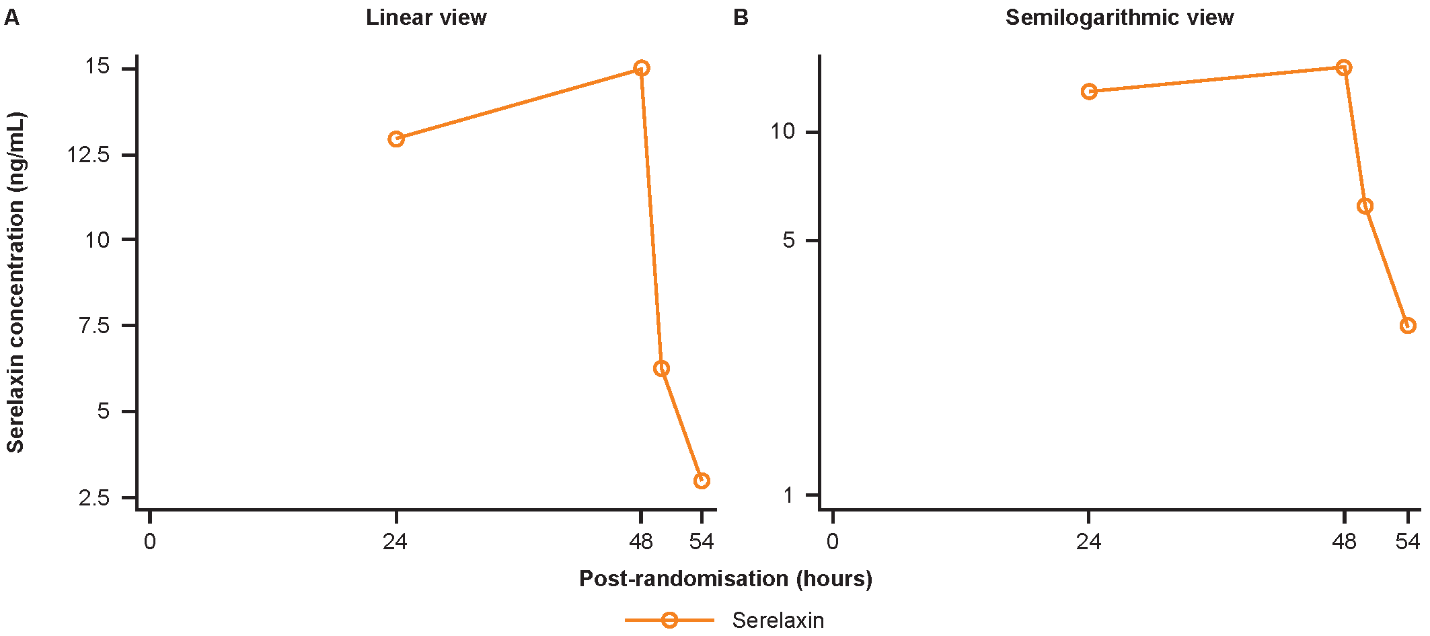
**

**Figure S6**

**
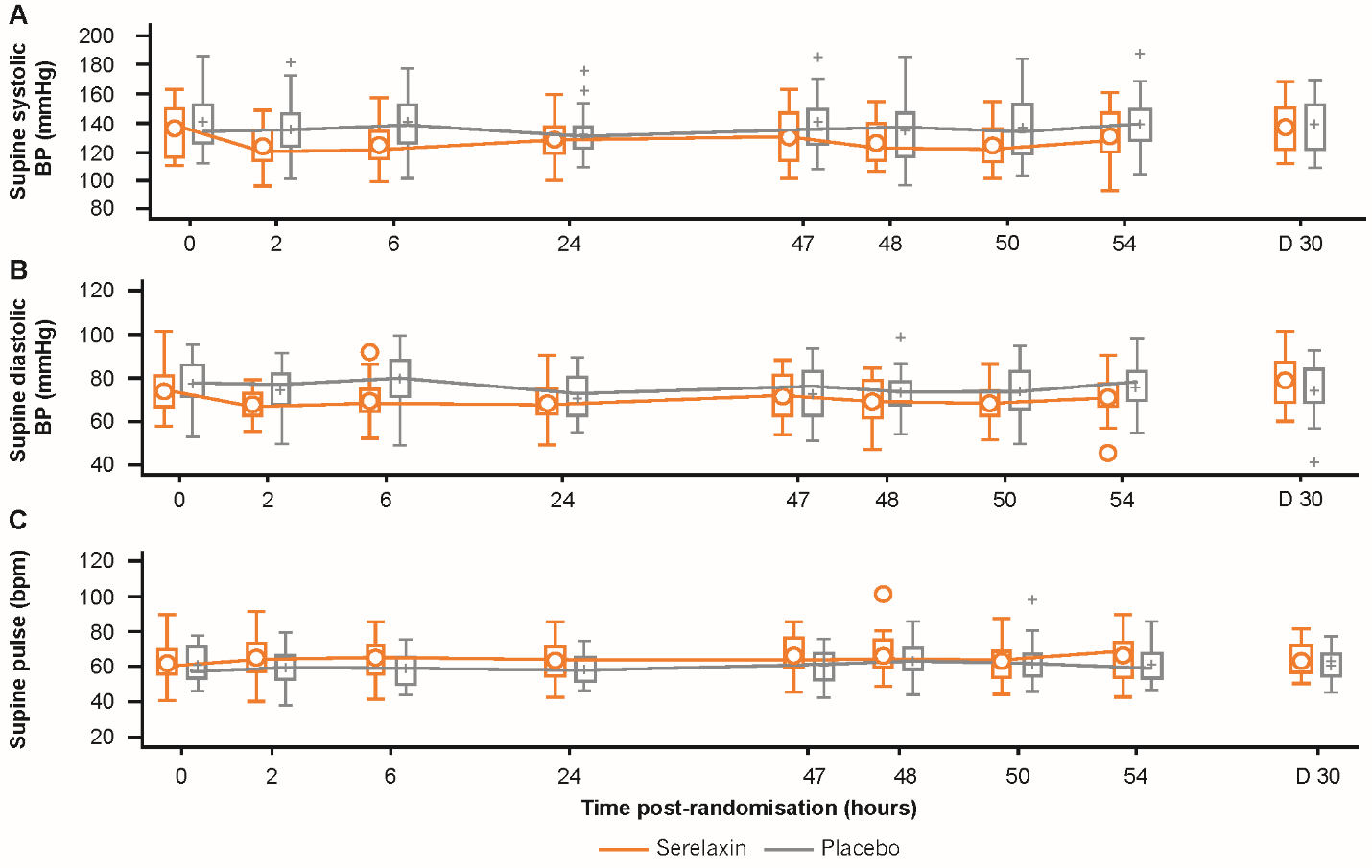
**

# Supplementary Tables

**Supplementary Table S1. Change from baseline in myocardial perfusion endpoints using repeated measures ANCOVA.**

| **Parameter (unit)** | **Treatment (*n*)** | **Adjusted mean difference** | **95% CI** | **Adjusted mean difference (serelaxin versus placebo)** | **95% CI** | ***p*-value** |
| --- | --- | --- | --- | --- | --- | --- |
| **Mean global MBF rest (mL/g/min)** | Serelaxin (22) | 0.01 | -0.08, 0.09 | 0.05 | -0.07, 0.17 | 0.40 |
|  | Placebo (25) | -0.05 | -0.13, 0.04 |  |  |  |
| **Mean global MBF stress (mL/g/min)** | Serelaxin (23) | -0.22 | -0.43, -0.01 | -0.05 | -0.34, 0.25 | 0.76 |
|  | Placebo (25) | -0.18 | -0.38, 0.03 |  |  |  |
| **Global MPR** | Serelaxin (22) | -0.24 | -0.45, -0.04 | -0.11 | -0.40, 0.18 | 0.44 |
|  | Placebo (25) | -0.13 | -0.33, 0.06 |  |  |  |
| **Mid MBF rest (mL/g/min)** | Serelaxin (22) | -0.01 | -0.10, 0.08 | 0.06 | -0.06, 0.19 | 0.30 |
|  | Placebo (25) | -0.08 | -0.16, 0.01 |  |  |  |
| **Mid MBF stress (mL/g/min)** | Serelaxin (23) | -0.27 | -0.51, -0.02 | -0.11 | -0.45, 0.22 | 0.50 |
|  | Placebo (25) | -0.15 | -0.38, 0.08 |  |  |  |
| **Mid perfusion reserve index** | Serelaxin (22) | -0.26 | -0.52, -0.01 | -0.19 | -0.54, 0.16 | 0.28 |
|  | Placebo (25) | -0.08 | -0.31, 0.16 |  |  |  |

*n*=the number of subjects used in the analysis at each time-point. CI, confidence interval; MBF, myocardial blood flow; MPR, myocardial perfusion reserve index.

**Supplementary Table S2.** **Absolute values for myocardial perfusion endpoints from cardiac MRI.**

| **Parameter (unit)** | **Hours post dose** | **Serelaxin (*n*)** | **Placebo (*n*)** |
| --- | --- | --- | --- |
| **Mean global MBF rest (mL/g/min)** | 0 | 1.26 ± 0.37 (22) | 1.19 ± 0.31 (25) |
|  | 47 | 1.24 ± 0.32 (24) | 1.16 ± 0.26 (25) |
| **Mean global MBF stress (mL/g/min)** | 0 | 2.54 ± 0.87 (24) | 2.34 ± 0.61 (25) |
|  | 47 | 2.32 ± 0.67 (23) | 2.21 ± 0.67 (25) |
| **Global MPR** | 0 | 2.25 ± 0.88 (22) | 2.17 ± 0.64 (25) |
|  | 47 | 1.98 ± 0.59 (23) | 2.06 ± 0.55 (25) |
| **Mid MBF rest (mL/g/min)** | 0 | 1.22 ± 0.37 (22) | 1.15 ± 0.33 (25) |
|  | 47 | 1.17 ± 0.31 (24) | 1.09 ± 0.22 (25) |
| **Mid MBF stress (mL/g/min)** | 0 | 2.45 ± 0.84 (24) | 2.23 ± 0.64 (25) |
|  | 47 | 2.16 ± 0.70 (23) | 2.14 ± 0.65 (25) |
| **Mid perfusion reserve index** | 0 | 2.24 ± 0.98 (22) | 2.08 ± 0.66 (25) |
|  | 47 | 1.93 ± 0.70 (23) | 2.05 ± 0.65 (25) |
| **Apical MBF rest (mL/g/min)** | 0 | 1.41 ± 0.43 (22) | 1.32 ± 0.31 (25) |
|  | 47 | 1.41 ± 0.34 (24) | 1.31 ± 0.27 (25) |
| **Apical MBF stress (mL/g/min)** | 0 | 2.82 ± 1.00 (24) | 2.65 ± 0.71 (25) |
|  | 47 | 2.66 ± 0.73 (23) | 2.44 ± 0.67 (25) |
| **Apical perfusion reserve index** | 0 | 2.24 ± 0.89 (22) | 2.20 ± 0.77 (25) |
|  | 47 | 2.01 ± 0.60 (23) | 1.99 ± 0.53 (25) |
| **Basal MBF rest (mL/g/min)** | 0 | 1.16 ± 0.39 (22) | 1.09 ± 0.33 (25) |
|  | 47 | 1.13 ± 0.39 (24) | 1.07 ± 0.34 (25) |
| **Basal MBF stress (mL/g/min)** | 0 | 2.34 ± 0.90 (24) | 2.15 ± 0.84 (25) |
|  | 47 | 2.14 ± 0.84 (23) | 2.05 ± 0.94 (25) |
| **Basal perfusion reserve index** | 0 | 2.25 ± 0.92 (22) | 2.19±0.62 (24) |
|  | 47 | 1.10 ± 0.68 (23) | 2.17±0.66 (23) |

The observed arithmetic mean values for all the myocardial perfusion endpoints from cardiac MRI myocardial blood flow on day 3, 47 h were comparable between serelaxin and placebo. Values are presented as mean ± SD unless otherwise specified. *n*=the number of subjects used in the analysis at each time-point.

MBF, myocardial blood flow; MPR, myocardial perfusion reserve; MRI, magnetic resonance imaging.

**Supplementary Table S3.** **Change from baseline in myocardial perfusion endpoints using Bayesian analysis.**

| **Parameter** | **Treatment (*n*)** | **Within treatment** | | **Estimate of difference** | | **Probability of success** | |
| --- | --- | --- | --- | --- | --- | --- | --- |
|  |  | **Posterior median** | **95% Cred. int.** | **Posterior median** | **95% Cred. int.** | **Pr (Criterion 1\|data)*** | **Pr (Criterion 2\|data)#** |
| **Mean global MBF rest (mL/g/min)** | Serelaxin (22) | 0.01 | (-0.08, 0.09) | 0.05 | (-0.07, 0.17) | 0.80 | 0.0014 |
|  | Placebo (25) | -0.05 | (-0.13, 0.04) |  |  |  |  |
| **Mean global MBF stress (mL/g/min)** | Serelaxin (23) | -0.22 | (-0.43, 0.01) | -0.05 | (-0.35, 0.25) | 0.38 | 0.0006 |
|  | Placebo (25) | -0.17 | (-0.37, 0.03) |  |  |  |  |
| **Global MPR*** | Serelaxin (22) | -0.25 | (-0.46, 0.03) | -0.11 | (-0.40, 0.19) | 0.22 | 0.0002 |
|  | Placebo (25) | -0.13 | (-0.33, 0.07) |  |  |  |  |

Global myocardial blood flow (Global MBF): Derived from the kinetic analysis of the first pass myocardial perfusion CMR images acquired at rest and under adenosine stress conditions. Myocardial images are analysed with the 16 segment American Heart Association (AHA) model and the global blood flow value is the mean over all segments.

Myocardial perfusion reserve (MPR): *Ratio of the global blood flow values, global MBF stress / global MBF rest

The change from baseline to day 3 was analysed using Bayesian approach assuming non-informative priors.

*criterion 1: Increase in MPRI between serelaxin and placebo is greater than zero

#criterion 2: Increase in MPRI between serelaxin and placebo is greater than 20%

CMR, cardiovascular magnetic resonance imaging; Cred.int., Bayesian credible interval; MBF, myocardial blood flow; MPR, myocardial perfusion reserve; Pr, probability of success.

**Supplementary Table S4. Change from baseline in augmentation index (AIx) using repeated measures ANCOVA.**

| **Parameter (unit)** | **Time** | **Treatment (*n*)** | **Adjusted mean difference** | **95% CI** | **Adjusted mean difference (serelaxin versus placebo)** | **95% CI** | ***p*-value** |
| --- | --- | --- | --- | --- | --- | --- | --- |
| **AIx (%)** | 2 h | Serelaxin (23) | -4.09 | -7.97,-0.21 | -2.12 | -7.49, 3.26 | 0.43 |
|  |  | Placebo (25) | -1.97 | -5.69, 1.75 |  |  |  |
|  | 6 h | Serelaxin (24) | -3.28 | -6.13,-0.42 | -2.88 | -6.84, 1.08 | 0.15 |
|  |  | Placebo (26) | -0.39 | -3.14, 2.35 |  |  |  |
|  | 24 h | Serelaxin (24) | -0.77 | -4.02, 2.47 | -0.84 | -5.38, 3.70 | 0.71 |
|  |  | Placebo (25) | 0.07 | -3.11, 3.25 |  |  |  |
|  | 47 h | Serelaxin (23) | 3.49 | -0.47, 7.44 | 3.45 | -2.04, 8.95 | 0.21 |
|  |  | Placebo (25) | 0.04 | -3.78, 3.85 |  |  |  |
|  | 50 h | Serelaxin (22) | -0.76 | -5.21, 3.68 | 3.25 | -2.90, 9.41 | 0.29 |
|  |  | Placebo (24) | -4.02 | -8.27, 0.24 |  |  |  |
|  | 54 h | Serelaxin (22) | -1.74 | -5.92, 2.44 | -0.98 | -6.72, 4.76 | 0.73 |
|  |  | Placebo (25) | -0.75 | -4.69, 3.18 |  |  |  |
|  | Day 30 | Serelaxin (25) | -0.98 | -4.91, 2.95 | -1.76 | -7.27, 3.75 | 0.53 |
|  |  | Placebo (26) | 0.78 | -3.08, 4.63 |  |  |  |
|  | Day 180 | Serelaxin (24) | 0.23 | -3.07, 3.54 | -0.05 | -4.67, 4.57 | 0.98 |
|  |  | Placebo (25) | 0.28 | -2.95, 3.52 |  |  |  |

*n*=the number of subjects used in the analysis at each time-point.

AIx, augmentation index; CI, confidence interval; h, hours

**Supplementary Table S5. Absolute values for augmentation index (AIx).**

| **Parameter (unit)** | **Time post-dose** | **Serelaxin (*n*)** | **Placebo (*n*)** |
| --- | --- | --- | --- |
| **AIx (%)** | 0 h | 25.81 ± 7.70 (25) | 26.31 ± 11.17 (26) |
|  | 2 h | 22.17 ± 10.09 (23) | 24.32 ± 9.06 (25) |
|  | 6 h | 22.96 ± 6.95 (24) | 25.73 ± 8.23 (26) |
|  | 24 h | 25.08 ± 8.10 (24) | 26.04 ± 10.23 (25) |
|  | 47 h | 29.57 ± 9.75 (23) | 26.04 ± 10.04 (25) |
|  | 50 h | 24.89 ± 12.50 (22) | 22.29 ± 10.67 (24) |
|  | 54 h | 24.70 ± 10.62 (22) | 25.34 ± 9.35 (25) |
|  | Day 30 | 24.98 ± 9.05 (25) | 26.88 ± 11.01 (26) |
|  | Day 180 | 26.15 ± 8.97 (24) | 26.36 ± 8.98 (25) |

Mean of AIx in serelaxin was comparable for all time points when compared with placebo. Values are presented as mean ± SD unless otherwise specified.

*n*=the number of subjects used in the analysis at each time-point.

AIx, augmentation index; h, hours.

**Supplementary Table S6. Change from baseline in pulse wave velocity (PWV) using repeated measures ANCOVA.**

| **Parameter (unit)** | **Time** | **Treatment (*n*)** | **Adjusted mean difference** | **95% CI** | **Adjusted mean difference**  **(serelaxin versus placebo)** | **95% CI** | **Two-sided *p-*value*** |
| --- | --- | --- | --- | --- | --- | --- | --- |
| **PWV (meters/second)** | 24 h | Serelaxin (19) | -0.54 | -1.06, -0.03 | -0.157 | -0.856, 0.543 | 0.65 |
|  |  | Placebo (24) | -0.39 | -0.85, 0.08 |  |  |  |
|  | 47 h | Serelaxin (21) | -0.26 | -0.90, 0.38 | -0.34 | -1.24, 0.56 | 0.45 |
|  |  | Placebo (23) | 0.08 | -0.53, 0.70 |  |  |  |
|  | Day 30 | Serelaxin (20) | 0.35 | -0.31, 1.02 | -0.06 | -0.97, 0.86 | 0.90 |
|  |  | Placebo (24) | 0.41 | -0.21, 1.03 |  |  |  |
|  | Day 180 | Serelaxin (20) | 0.93 | 0.24, 1.62 | 0.12 | -0.82, 1.06 | 0.80 |
|  |  | Placebo (24) | 0.81 | 0.18, 1.44 |  |  |  |

*Two-sided *p*-value is testing for difference (*p*-value < 0.05) is considered as statistically significant

*n*=the number of subjects used in the analysis at each time-point.

CI, confidence interval; h, hours; PWV, pulse wave velocity

**Supplementary Table S7. Absolute values for pulse wave velocity (PWV).**

| **Parameter (unit)** | **Time post-dose** | **Serelaxin** (***n*)** | **Placebo (*n*)** |
| --- | --- | --- | --- |
| **PWV (meters/second)** | 0 h | 7.45 ± 2.05 (22) | 8.17 ± 1.95 (25) |
|  | 24 h | 7.05 ± 1.88 (19) | 7.68 ± 2.14 (24) |
|  | 47 h | 7.20 ± 1.92 (22) | 8.26 ± 2.50 (23) |
|  | Day 30 | 7.99 ± 1.82 (23) | 8.46 ± 2.44 (24) |
|  | Day 180 | 8.34 ± 2.11 (23) | 8.84 ± 2.17 (24) |

No systematic drug-induced change was observed. Values are presented as mean ± SD unless otherwise specified.

*n*=the number of subjects used in the analysis at each time-point.

h, hours; PWV, pulse wave velocity.

**Supplementary Table S8A. Summary table of measurements of arterial stiffness from aortic MRI.**

| **Parameter (unit)** | **Hours post-dose** | **Serelaxin (*n*)** | **Placebo (*n*)** |
| --- | --- | --- | --- |
| **Ascending aorta strain** | 0 | 0.12 ± 0.06 (24) | 0.11 ± 0.06 (22) |
|  | 47 | 0.12 ± 0.06 (23) | 0.11 ± 0.06 (22) |
| **Ascending aorta distensibility (mm/Hg)** | 0 | 0.002 ± 0.002 (24) | 0.001 ± 0.002 (22) |
|  | 47 | 0.002 ± 0.001 (23) | 0.001 ± 0.001 (22) |
| **Descending aorta strain** | 0 | 0.16 ± 0.06 (24) | 0.14 ± 0.06 (21) |
|  | 47 | 0.17 ± 0.05 (23) | 0.14 ± 0.07 (22) |
| **Descending aorta distensibility**  **(mm/Hg)** | 0 | 0.002 ± 0.001 (24) | 0.002 ± 0.002 (21) |
|  | 47 | 0.002 ± 0.001 (23) | 0.002 ± 0.001 (22) |
| **Peak flow velocity (cm/sec)** | 0 | 128.00 ± 66.69 (24) | 102.70 ± 33.90 (24) |
|  | 47 | 127.98 ± 60.76 (23) | 106.56 ± 38.96 (25) |

*n*=number of patients used in the analysis for each parameter. Values are presented as mean ± SD unless otherwise specified.

MRI, magnetic resonance imaging; SD, standard deviation.

**Supplementary Table S8B. Statistical analysis of change from baseline to day 3 in measurements of arterial stiffness from Aortic MRI using ANCOVA.**

| **Parameter (unit)** | **Treatment (*n*)** | **Adjusted mean difference** | **(95% CI)** | **Adjusted mean difference**  **(serelaxin versus placebo)** | **(95% CI)** | **Two-sided *p*-value*** |
| --- | --- | --- | --- | --- | --- | --- |
| **Ascending aorta strain** | Serelaxin (23) | 0.01 | (-0.01, 0.02) | 0.01 | (-0.10, 0.03) | 0.31 |
|  | Placebo (22) | -0.004 | (-0.02, 0.01) |  |  |  |
| **Ascending aorta distensibility (mm/Hg)** | Serelaxin (23) | -0.0001 | (-0.0004, 0.0002) | -0.0001 | (-0.0005, 0.0004) | 0.74 |
|  | Placebo (22) | -0.00 | (-0.0003, 0.0003) |  |  |  |
| **Descending aorta strain** | Serelaxin (23) | 0.011 | (-0.01, 0.03) | 0.01 | (-0.02, 0.03) | 0.61 |
|  | Placebo (21) | 0.005 | (-0.01, 0.02) |  |  |  |
| **Descending aorta distensibility (mm/Hg)** | Serelaxin (23) | 0.0001 | (-0.0003, 0.0005) | -0.0001 | (-0.0007, 0.0004) | 0.64 |
|  | Placebo (21) | 0.0002 | (-0.0002, 0.0006) |  |  |  |
| **Peak flow velocity (cm/sec)** | Serelaxin (23) | 10.12 | (-10.27, 30.51) | 13.71 | (-15.15, 42.57) | 0.34 |
|  | Placebo (24) | -3.60 | (-23.55, 16.36) |  |  |  |

*n*=number of patients used in the analysis for each parameter. For analysis of change from baseline, only subjects with results at both baseline and post-baseline can be included.

Model: Change from baseline was analysed using ANCOVA model including treatment as fixed effect and baseline as continuous covariate.

*Two-sided *p*-value is testing for difference (*p*-value <0.05) is considered as statistically significant. CI, confidence interval; MRI, magnetic resonance imaging.

**Supplementary Table S9. Summary of blood (plasma) biomarkers.**

| **Parameter (unit)** | **Time** | **Statistics** | **Serelaxin**  **(*N*=30)** | **Placebo**  **(*N*=28)** | **Change from baseline** | |
| --- | --- | --- | --- | --- | --- | --- |
|  |  |  |  |  | **Serelaxin (*N*=30)** | **Placebo**  **(*N*=28)** |
| **NT-proBNP**  **(pg/mL)** | 0 h | *n* | 29 | 26 |  |  |
|  |  | Geo-mean (95% CI) | 87.0 (60.2,  125.8) | 81.5 (54.9,  121.1) |  |  |
|  | 24 h | *n* | 27 | 26 | 27 | 26 |
|  |  | Geo-mean (95% CI) | 74.7 (51.9,  107.5) | 63.5 (43.1, 93.5) | 0.89 (0.77, 1.02) | 0.78 (0.69,  0.88) |
|  | 48 h | *n* | 28 | 26 | 28 | 26 |
|  |  | Geo-mean (95% CI) | 86.8 (57.7,  130.5) | 61.6 (37.4,  101.5) | 1.03 (0.79, 1.34) | 0.76 (0.60,  0.95) |
|  | 54 h | *n* | 27 | 25 | 27 | 25 |
|  |  | Geo-mean (95% CI) | 117.1 (79.9,  171.7) | 83.3 (54.4,  127.5) | 1.36 (1.06, 1.75) | 0.91 (0.71,  1.15) |
|  | Day 30 | *n* | 29 | 25 | 29 | 25 |
|  |  | Geo-mean (95% CI) | 88.4 (60.3,  129.7) | 89.5 (54.5,  146.8) | 1.02 (0.80, 1.29) | 1.06 (0.77,  1.45) |
|  | Day 180 | *n* | 29 | 25 | 29 | 25 |
|  |  | Geo-mean (95% CI) | 101.6 (69.9,  147.8) | 95.1 (65.1,  139.1) | 1.17 (0.86, 1.58) | 1.22 (0.94,  1.60) |
| **hs Troponin-T (µg/mL)** | 0 h | *n* | 29 | 24 |  |  |
|  |  | Geo-mean (95% CI) | 0.0076 (0.0057,  0.0102) | 0.0071 (0.0054,  0.0091) |  |  |
|  | 24 h | *n* | 27 | 24 | 27 | 24 |
|  |  | Geo-mean (95% CI) | 0.0064 (0.0049,  0.0084) | 0.0066 (0.0050,  0.0088) | 0.90 (0.80, 1.01) | 0.94 (0.84,  1.06) |
|  | 48 h | *n* | 28 | 23 | 28 | 23 |
|  |  | Geo-mean (95% CI) | 0.0066 (0.0049,  0.0089) | 0.0076 (0.0053,  0.0109) | 0.87 (0.79, 0.95) | 1.03 (0.76,  1.39) |
|  | 54 h | *n* | 27 | 23 | 27 | 23 |
|  |  | Geo-mean (95% CI) | 0.0079 (0.0057,  0.0110) | 0.0082 (0.0054,  0.0125) | 1.02 (0.85, 1.24) | 1.12 (0.75,  1.65) |
|  | Day 30 | *n* | 29 | 23 | 29 | 23 |
|  |  | Geo-mean (95% CI) | 0.0068 (0.0053,  0.0088) | 0.0080 (0.0054,  0.0118) | 0.89 (0.78, 1.02) | 1.12 (0.78,  1.60) |
|  | Day 180 | *n* | 29 | 24 | 29 | 24 |
|  |  | Geo-mean (95% CI) | 0.0083 (0.0066,  0.0105) | 0.0077 (0.0062,  0.0094) | 1.09 (0.91, 1.31) | 1.08 (0.91,  1.29) |
| **Cystatin C (mg/L)** | 0 h | *n* | 29 | 26 |  |  |
|  |  | Geo-mean (95% CI) | 0.931 (0.876,  0.990) | 0.867 (0.806,  0.932) |  |  |
|  | 24 h | *n* | 27 | 26 | 27 | 26 |
|  |  | Geo-mean (95% CI) | 0.839 (0.788,  0.893) | 0.849 (0.782,  0.922) | 0.92 (0.89, 0.94) | 0.98 (0.93,  1.03) |
|  | 48 h | *n* | 28 | 26 | 28 | 26 |
|  |  | Geo-mean (95% CI) | 0.867 (0.812,  0.926) | 0.855 (0.795,  0.919) | 0.93 (0.91, 0.96) | 0.99 (0.95,  1.02) |
|  | 54 h | *n* | 27 | 23 | 27 | 23 |
|  |  | Geo-mean (95% CI) | 0.796 (0.721,  0.878) | 0.811 (0.751,  0.876) | 0.87 (0.80, 0.94) | 0.95 (0.91,  0.99) |
|  | Day 30 | *n* | 29 | 25 | 29 | 25 |
|  |  | Geo-mean (95% CI) | 0.899 (0.842,  0.960) | 0.880 (0.815,  0.951) | 0.97 (0.93, 1.00) | 1.01 (0.97,  1.05) |
|  | Day 180 | *n* | 29 | 26 | 29 | 26 |
|  |  | Geo-mean (95% CI) | 0.908 (0.821,  1.004) | 0.893 (0.846,  0.942) | 0.98 (0.89, 1.07) | 1.03 (0.99,  1.07) |
| **Endothelin-1 (pmol/L)** | 0 h | *n* | 29 | 23 |  |  |
|  |  | Geo-mean (95% CI) | 0.874 (0.630,  1.212) | 0.771 (0.571,  1.042) |  |  |
|  | 24 h | *n* | 27 | 23 | 27 | 23 |
|  |  | Geo-mean (95% CI) | 0.6732 (0.4626,  0.9796) | 0.8472 (0.6456,  1.1118) | 0.82 (0.68, 0.99) | 1.10 (0.95,  1.28) |
|  | 48 h | *n* | 28 | 23 | 28 | 23 |
|  |  | Geo-mean (95% CI) | 0.6835 (0.4647,  1.0054) | 0.8340 (0.6445,  1.0791) | 0.82 (0.70, 0.96) | 1.08 (0.92,  1.27) |
|  | 54 h | *n* | 26 | 22 | 26 | 22 |
|  |  | Geo-mean (95% CI) | 0.7535 (0.5098,  1.1138) | 0.8719 (0.6418,  1.1845) | 0.84 (0.73, 0.96) | 1.12 (0.94,  1.34) |
|  | Day 30 | *n* | 29 | 23 | 29 | 23 |
|  |  | Geo-mean (95% CI) | 0.8145 (0.5881,  1.1279) | 0.9173 (0.6054,  1.3900) | 0.93 (0.77, 1.13) | 1.19 (0.90,  1.56) |
|  | Day 180 | *n* | 29 | 23 | 29 | 23 |
|  |  | Geo-mean (95% CI) | 0.7664 (0.5436,  1.0806) | 0.9022 (0.6374,  1.2771) | 0.88 (0.69, 1.12) | 1.17 (0.92,  1.48) |

At each time point, only patients with a value at both baseline and that time point are included.

*n*=all non-missing observations, including values <LLOQ and >ULOQ.

Baseline corresponds to the value at day 1 pre-dose. For change from baseline, geometric mean and its CI are calculated for the value (post) to baseline ratio.

CI, confidence interval; h, hours; LLOQ, lower limit of quantification; ULOQ; upper limit of quantification.

**Supplementary Table S10. Statistical analysis of change from baseline in vital signs (BP) using repeated measures ANCOVA.**

| **Variable** | **Hours post-dose** | **Adjusted mean difference** | | **Serelaxin versus placebo (95% CI)** | **Two-sided *p*-value** |
| --- | --- | --- | --- | --- | --- |
|  |  | **Serelaxin** | **Placebo** |  |  |
| **Supine SBP (mmHg)** | 2 | -12.73 | -3.15 | -9.58  (-17.08, -2.08) | 0.01 |
|  | 6 | -12.09 | 1.42 | -13.5  (-20.42, -6.60) | 0.0003 |
|  | 24 | -8.88 | -6.13 | -2.75  (-10.47 4.97) | 0.48 |
|  | 47 | -6.22 | 1.14 | -7.36  (-15.28, 0.56) | 0.07 |
| **Supine DBP (mmHg)** | 2 | -7.07 | -1.88 | -5.20  (-9.45, -0.94) | 0.02 |
|  | 6 | -5.26 | 3.16 | -8.43  (-13.46, -3.40) | 0.002 |
|  | 24 | -6.34 | -5.35 | -0.98  (-5.78, 3.80) | 0.68 |
|  | 47 | -3.00 | -3.74 | 0.74  (-4.47, 5.94) | 0.78 |

CI, confidence interval; DBP, diastolic blood pressure; SBP, systolic blood pressure.

**Supplementary Table S11. Adverse events by preferred term*.**

| **Preferred term, n (%)** | **Serelaxin (*n*=30)** | **Placebo (*n*=28)** | **All patients (dosed; *n*=58)** |
| --- | --- | --- | --- |
| Subjects with at least one AE | 17 (57) | 19 (68) | 36 (62) |
| Headache | 2 (7) | 4 (14) | 6 (10) |
| Dizziness | 2 (7) | 2 (7) | 4 (6) |
| Haemoglobin decreased | 2 (7) | 2 (7) | 4 (6) |
| Angina pectoris | 1 (3) | 2 (7) | 3 (5) |
| Acute myocardial infarction | 2 (7) | 1 (4) | 2 (3) |
| Angina unstable | 1 (3) | 1 (4) | 2 (3) |
| Blood creatinine increased | 1 (3) | 1 (4) | 2 (3) |
| Dyspnoea | 2 (7) | 0 (0) | 2 (3) |
| Haematocrit decreased | 1 (3) | 1 (4) | 2 (3) |
| Musculoskeletal pain | 2 (7) | 0 (0) | 2 (3) |
| Presyncope | 0 (0) | 2 (7) | 2 (3) |

*n*=number of patients used in the analysis for each parameter.

*Only the AEs with an incidence of ≥ 2% are included in this table.
