# Supplementary figures and images for: Vascular effects of serelaxin in patients with stable coronary artery disease: a randomized placebo-controlled trial
Source: Cardiovasc Res. 2020 Feb 17;117(1):320–9. doi: 10.1093/cvr/cvz345 (PMC7797213; doi:10.1093/cvr/cvz345)

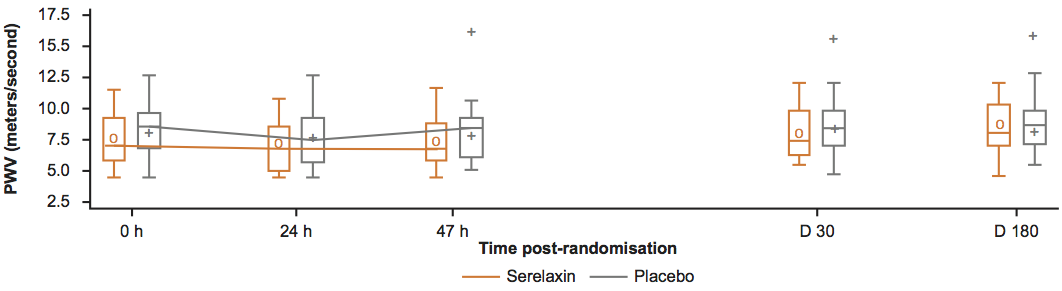

Supplement: cvz345_Supplementary_Data [file cvz345_supplementary_data.zip › cvz345-suppl_data/Supplemental Figure 1_TIFF.tiff]

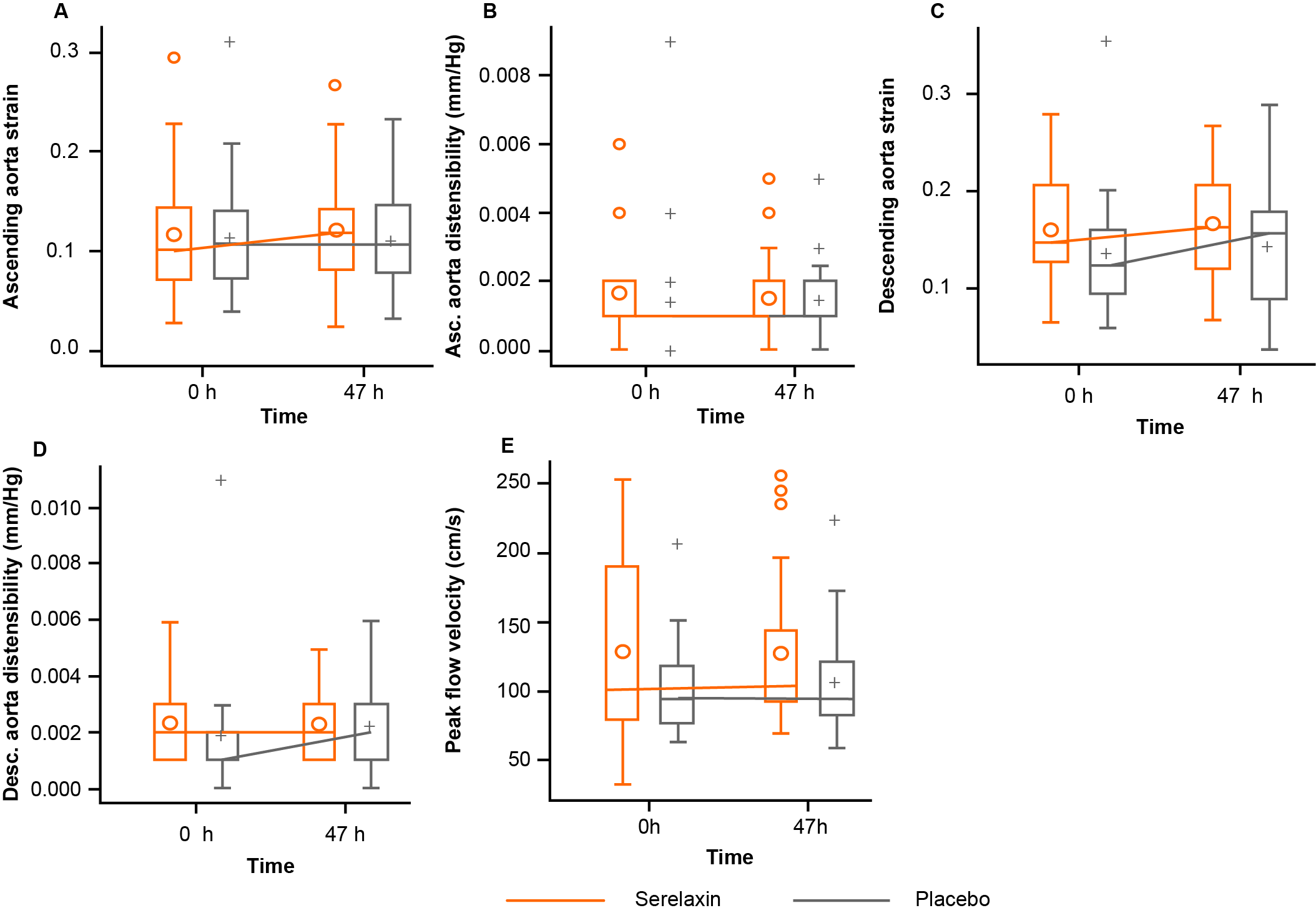

Supplement: cvz345_Supplementary_Data [file cvz345_supplementary_data.zip › cvz345-suppl_data/Supplemental Figure 2_TIFF.tif]

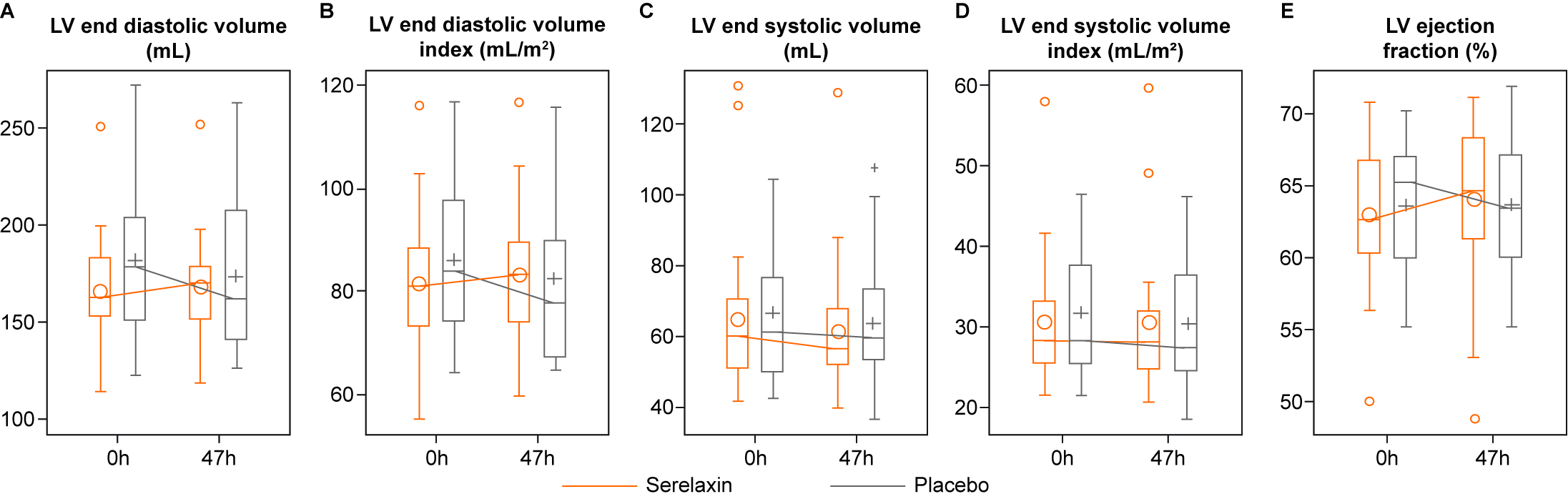

Supplement: cvz345_Supplementary_Data [file cvz345_supplementary_data.zip › cvz345-suppl_data/Supplemental Figure 3_TIFF.tif]

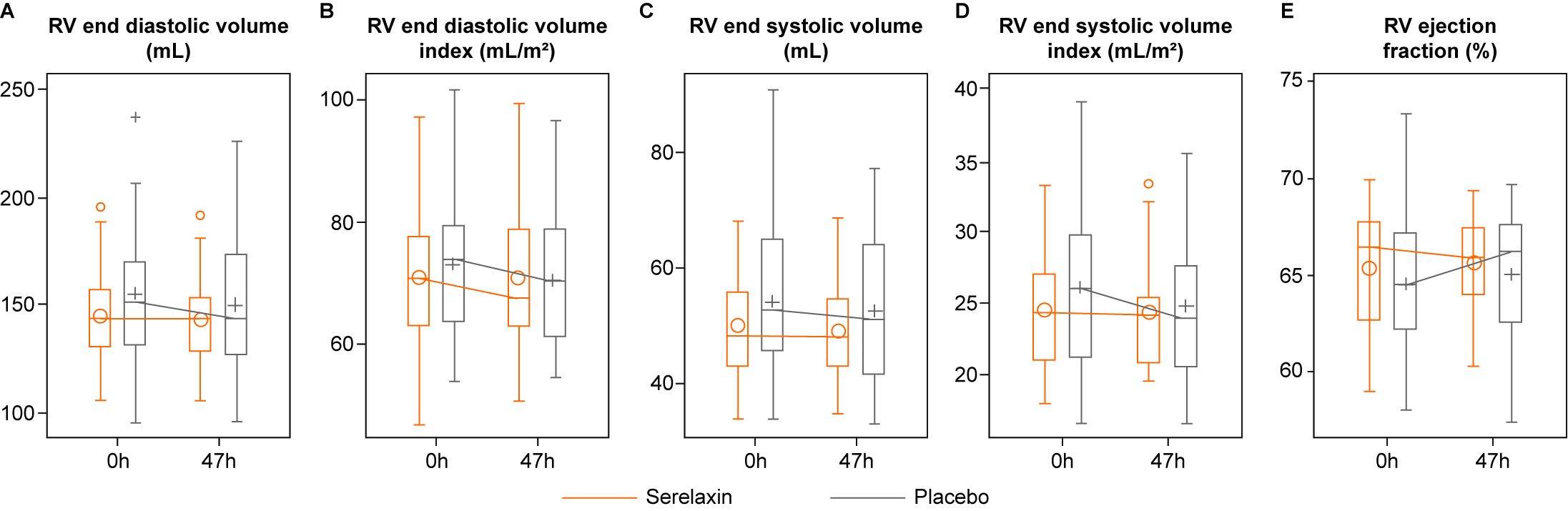

Supplement: cvz345_Supplementary_Data [file cvz345_supplementary_data.zip › cvz345-suppl_data/Supplemental Figure 4_TIFF.tif]

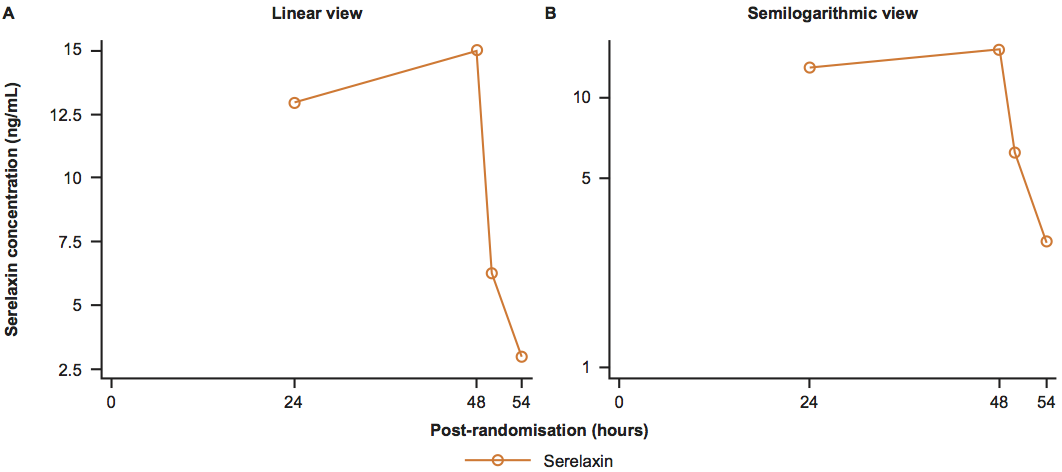

Supplement: cvz345_Supplementary_Data [file cvz345_supplementary_data.zip › cvz345-suppl_data/Supplemental Figure 5_TIFF.tiff]

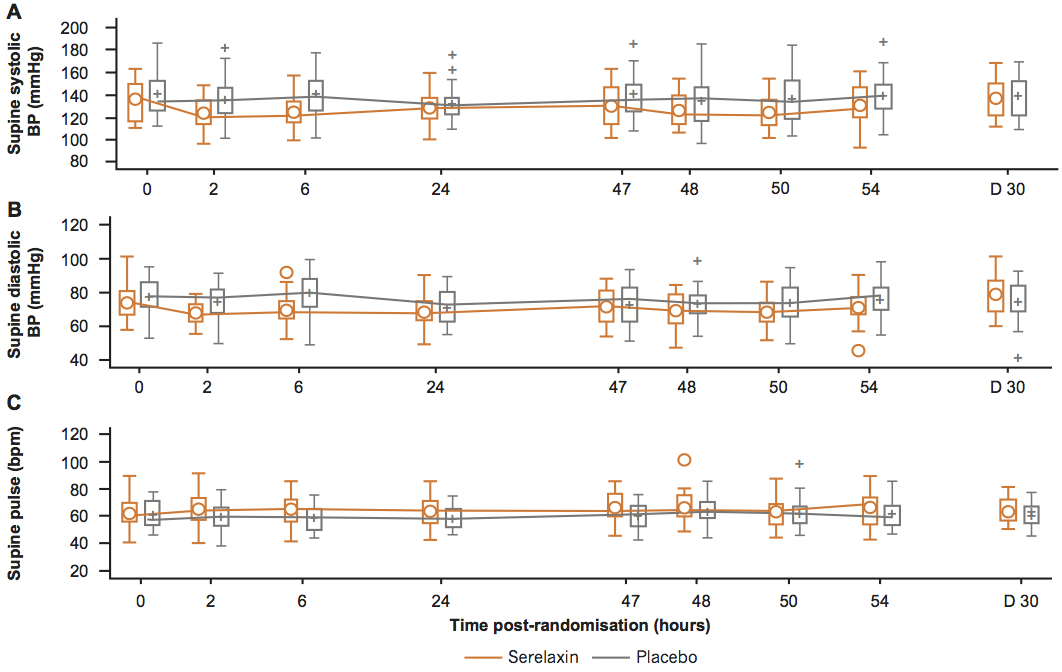

Supplement: cvz345_Supplementary_Data [file cvz345_supplementary_data.zip › cvz345-suppl_data/Supplemental Figure 6_TIFF.tiff]
